# A scoping review of network meta-analyses assessing the efficacy and safety of complementary and alternative medicine interventions

**DOI:** 10.1186/s13643-020-01328-3

**Published:** 2020-04-30

**Authors:** Misty Pratt, Susan Wieland, Nadera Ahmadzai, Claire Butler, Dianna Wolfe, Kusala Pussagoda, Becky Skidmore, Argie Veroniki, Patricia Rios, Andrea C. Tricco, Brian Hutton

**Affiliations:** 1 Clinical Epidemiology Program, Ottawa Hospital Research Institute, 501 Smyth Road, Box 201, Ottawa, Ontario K1H 8 L6 Canada; 2 University of Maryland School of Medicine, Baltimore, MD USA; 3 Department of Primary Education, School of Education, University of Ioannina, Ioannina, Greece; 4 Li Ka Shing Knowledge Institute, St Michael’s Hospital, Unity Health Toronto, Toronto, Canada; 5 Institute of Reproductive and Developmental Biology, Department of Surgery & Cancer, Faculty of Medicine, Imperial College, London, United Kingdom; 6 Epidemiology Division, Dalla Lana School of Public Health and Institute for Health Policy, Management, and Evaluation, University of Toronto, Toronto, Canada; 7 School of Epidemiology and Public Health, University of Ottawa, Ottawa, Canada

**Keywords:** Complementary and alternative medicine, Scoping review, Network meta-analysis

## Abstract

**Background:**

Network meta-analysis (NMA) has rapidly grown in use during the past decade for the comparison of healthcare interventions. While its general use in the comparison of conventional medicines has been studied previously, to our awareness, its use to assess complementary and alternative medicines (CAM) has not been studied. A scoping review of the literature was performed to identify systematic reviews incorporating NMAs involving one or more CAM interventions.

**Methods:**

An information specialist executed a multi-database search (e.g., MEDLINE, Embase, Cochrane), and two reviewers performed study selection and data collection. Information on publication characteristics, diseases studied, interventions compared, reporting transparency, outcomes assessed, and other parameters were extracted from each review.

**Results:**

A total of 89 SR/NMAs were included. The largest number of NMAs was conducted in China (39.3%), followed by the United Kingdom (12.4%) and the United States (9.0%). Reviews were published between 2010 and 2018, with the majority published between 2015 and 2018. More than 90 different CAM therapies appeared at least once, and the median number per NMA was 2 (IQR 1–4); 20.2% of reviews consisted of only CAM therapies. Dietary supplements (51.1%) and vitamins and minerals (42.2%) were the most commonly studied therapies, followed by electrical stimulation (31.1%), herbal medicines (24.4%), and acupuncture and related treatments (22.2%). A diverse set of conditions was identified, the most common being various forms of cancer (11.1%), osteoarthritis of the hip/knee (7.8%), and depression (5.9%). Most reviews adequately addressed a majority of the PRISMA NMA extension items; however, there were limitations in indication of an existing review protocol, exploration of network geometry, and exploration of risk of bias across studies, such as publication bias.

**Conclusion:**

The use of NMA to assess the effectiveness of CAM interventions is growing rapidly. Efforts to identify priority topics for future CAM-related NMAs and to enhance methods for CAM comparisons with conventional medicine are needed.

**Systematic review registration:**

https://ruor.uottawa.ca/handle/10393/35658

## Background

The use of complementary and alternative medicine (CAM) interventions is common [1–6], and the number of randomized controlled trials (RCT) and systematic reviews related to CAM interventions have previously been shown to be on the rise [7, 8]. As physicians are sometimes hesitant to discuss the use of CAM therapies with patients due to a lack of comfort in addressing related questions, there is a need to ensure rigorous scientific evidence of their benefits and harms is available [9, 10]. Past research has suggested that reviews of CAM interventions have been associated with certain areas of strength and weakness in terms of rigor relative to systematic reviews of other types of interventions [11], and challenges regarding clinical trial design and priority setting have also been identified [12].

Network meta-analysis (NMA) is a generalization of traditional pairwise meta-analysis [13, 14] and the use of NMA has grown rapidly in recent years [15–17]. NMA is of considerable value to researchers, analysts, and decision-makers when dealing with clinical scenarios requiring the comparison of multiple alternative therapies, as well as scenarios where there exists both direct and indirect evidence of relevance to the research question at hand [18, 19]. Methodologic research related to the conduct of NMA has also grown rapidly, and its use is also supported by helpful implementation tools including reporting guidance, overviews of adapted procedures for judging the strength of evidence, and published considerations for critical appraisal by decision-makers [20–23].

While the use of NMA for the comparison of pharmacologic interventions is common in the literature [16], the frequency of and approaches to its use for the evaluation of benefits and harms of complementary and alternative medicine (CAM) interventions—whether against each other or relative to other non-CAM interventions—to our awareness, has not been studied. In order to inform comparisons between traditional and complementary therapies, NMA represents a potentially valuable tool to establish relative benefits and harms. In the current study, we used a scoping review approach to establish the extent of published NMAs involving CAM interventions in the literature, to assess their objectives as well as clinical and methodologic characteristics, and to judge the current level of reporting transparency based upon criteria of the PRISMA Extension Statement for Network Meta-Analysis [23]. This information will be of value to establish what topics have been assessed in existing NMAs in the literature, thereby helping to prioritize both research topics as well as methodologic approaches for NMAs involving CAM interventions moving forward for interested physicians, decision-makers, and patients alike. Findings from this review will be informative for researchers and stakeholders seeking to prioritize future topics for CAM-related NMAs and may also allow for the identification of conditions wherein future randomized controlled trials of CAM interventions may be informative in the derivation of comparisons with traditional medical interventions.

## Review methods

A protocol for this review was drafted a priori by members of the authorship team. The protocol is available from the University of Ottawa Library’s Online Repository (available from https://www.ruor.uottawa.ca/handle/10393/35658). This review has been prepared in consideration of the guidance provided by the PRISMA extension statement for scoping reviews as well as the Joanna Briggs Institute [24, 25].

### Literature review, eligibility criteria and study selection

Published NMAs involving CAM interventions were identified for the current review using a combination of two approaches. First, three co-authors (AV, PR, ACT) maintained a database of all published NMAs published between 1999 and 2015 based upon a multi-database search strategy (including Medline, Embase, and the Cochrane Database) updated on a quarterly basis, with screening of citations and full texts performed by two independent reviewers; details of the search strategy used to establish the database are provided in Additional file 1. An update of the search was performed on May 29, 2018, with analogous techniques for screening of titles/abstracts and full-text articles used to identify and include relevant review articles. From the perspective of identifying reviews including NMA, studies selected for inclusion in the database were required to: (a) have used a valid comparison method (such as adjusted indirect comparison, Bayesian model, meta-regression, multivariate meta-analysis, graph theoretical approach); (b) included a minimum of 4 interventions in the network of evidence studied; (c) included a greater number of studies than there were nodes in the network; and (d) included data from RCTs only. For the purposes of the current review, studies identified from the above screening procedures were also reviewed in additional detail in terms of their included interventions to identify reviews that involved one or more CAM interventions; a listing of CAM therapies used during screening is provided in Additional file 2. Screening for reviews incorporating CAM interventions was performed by two independent reviewers (MP, SW). Articles which were focused upon statistical methods investigations relative to NMA were not included in the current review. The process of study selection was summarized using a flow diagram. Only English language reviews were included.

### Data collection procedures

A detailed list of information was gathered from each included study that met the study objectives. This information included publication characteristics (i.e., authorship list, year, and journal of publication), core features of each review (e.g., aspects of the research question addressed including study population and endpoints assessed, CAM therapies evaluated), characteristics of each review’s network geometry (including whether only CAM interventions were compared in isolation, or whether CAM and non-CAM interventions were established as comparators; and underlying numbers of studies and patients informing analysis); and statistical aspects of analyses performed (including choice of framework [Bayesian vs frequentist], assessment of the consistency assumption, and reporting of secondary measures of treatment effect). The completeness of reporting for each SR/NMA was assessed using the checklist from the PRISMA Extension Statement for NMA [23]. This checklist addresses the 27 core items included in the PRISMA Statement [26] and also addresses 5 additional items specific to the reporting of network meta-analyses (including methods and reporting of findings for each of network geometry inspection and assessment of the appropriateness of the consistency assumption, as well as presentation of a network diagram of the available evidence).

### Charting the data

A descriptive approach to summarize the core study characteristics was prepared, along with structured tables and figures to identify salient points of differences noted across studies. A heat map was generated to present the geographic distribution of published reviews (based upon affiliation of each study’s lead author), while a word cloud was prepared to assess the relative frequencies with which different CAM interventions were studied in the set of included NMAs. Trends over time in the number of NMAs published with regard to different clinical conditions were reviewed. Bar graphs were generated to evaluate the proportions of included studies adequately addressing individual items of the PRISMA NMA Checklist related to abstract and introduction, methods, results, discussion, and funding status, respectively. Changes in the completeness of reporting were also assessed by year of publication to establish whether the proportions of studies assessed to be of adequate reporting transparency or review methods were improving over time.

## Results

### Identified literature and general characteristics

**Fig. 1:**
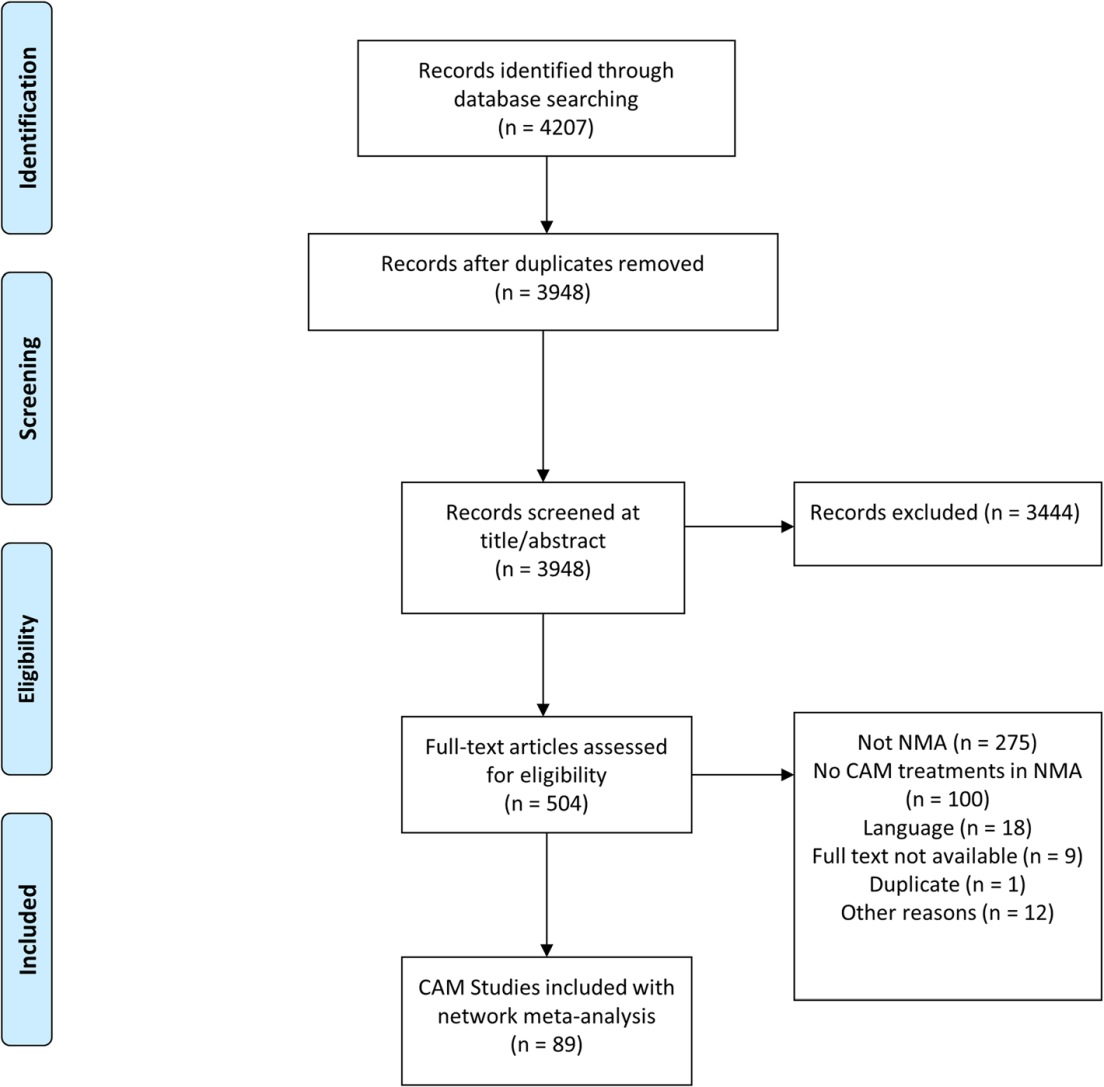
PRISMA flow diagram

In total, literature searching for this review identified a total of 3948 unique abstracts, 90 of which were retained as eligible network meta-analyses that included one or more CAM interventions according to the criteria described earlier [27–115]. Figure 1 presents a summary of the study selection process. Table 1 presents a detailed summary of the core characteristics of the included reviews, including patients’ indication, numbers of studies (and patients) analyzed, endpoints evaluated, key methods used, and review funding.

**Table 1:** Overview of included review articles

Author and year; country	Condition	Funding	# Included studies (patients)^*^	# Nodes in network^*^	Compared CAM with non-CAM?	CAM therapies studied?	Endpoints	Study designs included	Bayes (B) or frequentist (F) approach?	Consistency assumption addressed?	Provides a measurement of treatment ranking (e.g. SUCRA)?
Devoe (2018); Canada [44]	Clinical high risk for psychosis	Public	41 (3146)	7	Yes	Omega-3	Reduction of attenuated psychotic symptoms	RCTs; NRS	F	Yes	Yes
Di (2018); China [111]	Cerebral infarction	Public	37 (4330)	6	Yes	Ginkgo-dipyidamolum injections; Shuxuening injections; Ginaton injections; Ginkgolides injections; Floium Ginkgo extract and Tertram Ethypyrazine Sodium Chloride injections	Total effectiveness of cerebral infarction, changes of neural function defect score, ADRs/ADEs	RCTs	B	Yes	Yes
Feng (2018); China [58]	Insomnia	Public	20 (1339)	12	Yes	Acupuncture, language induction, listening to music, listening to music and acupuncture, listening to music and language induction, listening to placebo music, music-assisted relaxation, music-assisted relaxation and stimulus control, music with exercise, stimulus control	Sleep quality, sleep onset latency, sleep efficiency	RCTs; CCTs	B	Yes	Yes
Freeman (2018); UK [51]	Psychologic preparation for surgery under general anesthesia	Public	71 (NR)	16	Yes	Relaxation	Length of stay, postoperative pain, negative affect	RCTs	B	No	Yes
Fu (2018); China [50]	Spasticity in multiple sclerosis	Public	23 (2n720)	5	Yes	Transcutaneous electrical nerve stimulation	Spasticity scale, incidence of significant improvement, adverse effects	RCTs	B	No	Yes
Hilfiker (2018); Switzerland [54]	Cancer-related fatigue	Not reported	245 (NR)	12	Yes	Tai Chi, yoga, relaxation, dance, massage, music, healing touch	Cancer-related fatigue	RCTs; quasi-randomized	B	Yes	Yes
Khan (2018); Malaysia [71]	Stimulation of breast milk production	Public	5 (320)	6	No	Coleus amboinicus Lour (CA), fenugreek, palm dates	Milk production, maternal and neonatal safety	RCTs	F	No	No
Lee (2018); Korea [53]	Atrophic vaginitis	Public	9 (4034)	7	Yes	Sea buckthorn oil, soy isoflavone vaginal gel.	Efficacy for treatment of atrophic vaginitis and vaginal symptoms	RCTs	B	Yes	Yes
Liang (2018); China [70]	Alzheimer’s disease	Not reported	17 (1931)	4	Yes	Music therapy	Mini-Mental State Examination, Neuropsychiatric Inventory	RCTs	B	No	Yes
Pang (2018); China [56]	Prevention of type 2 diabetes (in patients with prediabetes)	Public	63 (8649)	11	Yes	Traditional Chinese medicine	Incidence of diabetes, regression to normoglycaemia	RCTs	F	No	Yes
Slade (2018); UK [45]	Bulemia nervosa	Public	21 (1828)	12	Yes	Relaxation	Full remission at the end of treatment	RCTs	B	Yes	Yes
Tsikopoulos (2018); Greece [52]	Chronic ankle instability	Not reported	19 (789)	13	Yes	Manual therapy	Foot and Ankle Ability Measure, Foot and Ankle Disability Index, Ankle Joint Functional Assessment Tool	RCTs	F	Yes	Yes
van den Akker (2018); Netherlands [47]	Pre-term birth adverse endpoints	No funding	51 (11,231)	26	No	Probiotics	Morbidity, mortality, necrotizing enterocolitis, late-onset sepsis, time until full enteral feeding	RCTs	B	Yes	No
Wei (2018); China [49]	Macular degeneration	Not reported	22 (2482)	11	Yes	Lutein; antioxidant complex, zinc-monocysteine, and a-lipoic acid	Best-corrected visual acuity change in GA area	RCTs	F	Yes	Yes
Xie (2018); China [55]	Kashin-Beck disease	Public	15 (2931)	7	No	Sodium selenite, selenium salt, sodium selenite with vitamin C, sodium selenite with vitamin E, selenium-enriched yeast, vitamin C	Effectiveness of selenium supplementation for the treatment of Kashin-Beck disease	RCTs	F	Yes	Yes
Zhang (2018); China [46]	Stable angina	Public	43 (4458)	5	Yes	Danhong, Danshen, salvianolate, compound Danshen	Clinical improvement rate	RCTs	B	Yes	No
Zhang (2018); China [107]	Gastric cancer	Public	26 (2154)	10	No	Aidi injection, Astragalus polysaccharide injection, capecitabine, China Biology Medicine disc, Compound Kushen injection, Disodium cantharidinate and vitamin B6 injection, Elemene injection, Huachansu injection, Javanica oil emulsion injection, Kangai injection, Lentinan injection, Shenfu injection, Shenmai injection, Shenqifuzheng injection, Xiaoaiping injection	Clinical effectiveness rate, performance status, ADRs	RCTs	B	No	Yes
Zhu (2018); China [42]	Osteoarthritis (hip, knee)	Public	61 (22,128)	6	Yes	Glucosamine, chondroitin	Pain intensity, function improvement and stiffness score, safety	RCTs	B	Yes	Yes
Kasatpibal (2017); Thailand [77]	Post-operative complications	Public	31 (2952)	7	Yes	Probiotics, prebiotics, synbiotics	SSI, UTI, pneumonia, sepsis, duration of antibiotic administration, length of hospital stay, mortality	RCTs	B	Yes	Yes
Amaral (2017); Brazil [87]	Prevention of respiratory tract infection	Public	22 (6603)	11	No	*Lactobacillus casei rhamnosus*, *Lactobacillus rhamnosus* T cell-1, *Lactobacillus reuteri*, *Bifidobacterium lactis*, *Lactobacillus rhamnosus* GG, *Lactobacillus fermentum* CECT5716, *Streptococcus salivarius* K12, *Bacillus clausii*	Respiratory tract infections, adverse effects	RCTs	B	Yes	Yes
Cai (2017); China [116]	Antibiotic-associated diarrhea	No funding	51 (9569)	9	No	Probiotics	Diarrhea, treatment tolerability and efficacy, C difficile infection rate, fever rate, dehydration rate	RCTs	F	Yes	Yes
Catala´-Lopez (2017); Canada [80]	Attention deficit hyperactivity disorder	Public	190 (26,114)	12	Yes	Ginkgo biloba, ginseng, pine bark extract, homeopathy, hypercium, iron, zinc, L-carcitine, minerals, amino acids, PUFA, omega3/6, herbal therapy	Treatment response, all-cause discontinuation, discontinuation due to adverse events, serious adverse events, specific adverse events	RCTs	B	Yes	No
Feng (2017); China [65]	Helicobacter pylori infection	Not reported	29 (3122)	12	No	Probiotics	H. pylori eradication rates, total side effects	RCTs	F	No	Yes
Feng (2017); China [67]	Crohn’s disease recurrence	Public	14 (877)	12	Yes	*Tripterygium wilfordii*, Lactobacillus GG	Endoscopic recurrence	RCTs	B	Yes	Yes
Fu (2017); China [66]	Neurotoxicity from chemotherapy	Public	23 (2869)	5	Yes	Calcium, magnesium, vitamin E	Overall neurotoxicity, severe neurotoxicity	RCTs	B	Yes	Yes
Haggman-Henrikson (2017); Sweden [63]	Chronic oro-facial pain	No funding	13 (1243)	6	Yes	Ping On	Pain intensity	RCTs	F	No	Yes
Han (2017); China [102]	Post-stroke recovery	Not reported	28 (2780)	7	No	Chinese Herbal Medicine: Dengzhan Shengmai, Gegensu, Huangqi plus Luotai, Huatuo Zaizao, MLC601 (NeuroAiD), Naoan, Naomaitai, Shuxuetong, Tongxinluo, Xueshuantong, Xixiantongshuan, Buchang Naoxintong, Chuanqiongqin, Mailuoning, Peiyuantongnao, Shenmai, Xuesaitong, Buchang Naoxintong plus Danhong Injection	Treatment response, neurological functional defect scores, Barthel index, Fugl–Meyer assessment, functional independence measure	RCTs	F	Yes	Yes
Ho (2017); Hong Kong [64]	Functional dyspepsia	Not reported	22 (1727)	11	Yes	Manual acupuncture, electroacupuncture	Alleviation of dyspeptic symptoms, % of patients achieving satisfactory alleviation of global or individual symptoms	SRs	F	Yes	Yes
Khaing (2017); Thailand [62]	Preeclampsia and gestational hypertension	No funding	27 (10,625)	4	No	Calcium, Vitamin D	Preeclampsia, eclampsia, gestational hypertension or pregnancy induced hypertension	RCTs	F	Yes	Yes
Li (2017); China [72]	Myofascial pain syndrome	Public	33 (1692)	10	Yes	Dry needling and muscle energy technique, scraping+warming acupuncture+moxibustion (SWAM), eletcro-acupuncture, manual acupuncture, electrospoon needle-cupping, dry needling and stretching, mini scalpel needle, multiple deep intramuscular stimulation therapy, sparrow-pecking, Myofascial trigger therapy, physical therapy	Pain intensity, pressure pain threshold, adverse events	RCTs	F	Yes	Yes
Ma (2017); China [113]	Gastrointestinal cancer	Public	23 (10,684)	9	Yes	Polysaccharide K	Overall survival, disease-free survival	RCTs	F	Yes	Yes
MacPherson (2017); USA [103]	Osteoarthritis (knee)	Public	114 (9709)	22	Yes	Acupuncture, tai chi, balneotherapy, TENS, pulsed electromagnetic field therapy, pulsed electrical stimulation, NMES	Pain	RCTs	B	Yes	Yes
Muñoz FSS (2017); Brazil [106]	Alzheimer's disease	Not reported	27 (4556)	7	No	Antioxidants, B-vitamins, inositol, medium-chain triglyceride, omega-3,polymeric formulas, polypeptide, vitamin D	Behavioral disturbances, cognitive/ functional/ global performance	RCTs	B	Yes	Yes
Sarri (2017); UK [57]	Vasomotor symptoms	Public	47 (8326)	7	Yes	Acupuncture, relaxation, multi-botanicals, valerin root, Chinese herbal medicine, black cohosh	Frequency of vasomotor symptoms at end of treatment, vaginal bleeding, treatment discontinuation	RCTs	B	Yes	Yes
Sekercioglu (2017); Canada [86]	Chronic kidney disease mineral and bone disorder	No funding	26 (6760)	8	Yes	Calcium, iron, calcium/magnesium	Phosphate levels; serum calcium; serum parathyroid hormone	RCTs	B	Yes	Yes
Su (2017); China [79]	Contrast-induced acute kidney injury	Public	150 (31,631)	12	Yes	N-acetylcysteine, vitamin and its analogues	Occurrence of contrast-induced acute kidney injury	RCTs	B	Yes	Yes
van Nooten (2017); Netherlands [84]	Diabetic neuropathy	Private	25 (5870)	6	Yes	Capsaicin 8% patch	% of patients with ≥ 30% and ≥ =50% pain reduction relative to baseline	RCTs	B	Yes	Yes
Wang (2017); China [68]	Helicobacter pylori infection	Public	140 (20,215)	7	No	Probiotics	Rates of eradication, adverse events	RCTs	B	Yes	Yes
Wang (2017); China [85]	Chronic fatigue syndrome	Public	31 (2255)	5	Yes	Chinese herbal medicine, acupuncture, moxibustion	Response rate	RCTs	B	Yes	Yes
Wei (2017); China [112]	Prevention of oxaliplatin-induced peripheral neurotoxicity (OIPN)	Public	25 (1572)	6	Yes	Huangqi injection, Shenmai injection, Shenfu injection, Buyang Huanwu decoction, Huangqi Guizhi Wuwu decoction	Overall OIPN incidence, severe OIPN incidence	RCTs	B	Yes	Yes
Wen (2017); China [61]	Helicobacter pylori infection	Not reported	17 (1932)	9	No	Probiotics	Eradication rates of H.pylori, side effects	RCTs	F	No	No
Westby (2017); UK [81]	Pressure ulcers	Public	51 (2947)	21	Yes	Honey-based wound dressing	Complete wound healing, time to complete healing	RCTs	B	Yes	Yes
Woods (2017); UK [78]	Osteoarthritis (knee)	Public	88 (7507)	18	Yes	Manual therapy, acupuncture, TENS, Balneotherapy, NMES, Tai Chi, PEMF, inferential therapy	Quality of life	RCTs	B	Yes	No
Yang (2017); China [69]	Blood pressure reduction	Public	19 (1459)	7	Yes	Qigong, tai chi, yoga	Systolic blood pressure, diastolic blood pressure	RCTs	B	Yes	Yes
Yeh (2017); China [59]	Psoriasis	Not reported	10 (1060)	6	Yes	Catgut embedding, acupuncture, acupressure, bloodletting	Treatment response, adverse events	RCTs	B	Yes	No
Yu (2017); China [83]	Necrotizing enterocolitis (NEC)	No funding	27 (4649)	4	Yes	Probiotics, arginine, lactoferrin, probiotics + fructo-oligosaccharides	NEC, all-cause mortality, sepsis, NEC-related mortality, hospitalization days	RCTs	B	No	Yes
Zhang (2017); China [105]	Gastric cancer	Public	81 (5978)	10	Yes	Chinese herbs injections	Treatment response, performance status, ADRs	RCTs	B	No	Yes
Zhang (2017); China [82]	Pancreatic cancer	Public	22 (1329)	8	Yes	Disodium cantharidinate and vitamin B6, Huanchansu, Javanica oil emulsion injection, Kangai, Kanglaite, Shenqifuzheng	Clinical effectiveness rate, performance status, nausea and vomiting, ADRs	RCTs	B	No	Yes
Zhang (2017); China [88]	Interstitial cystitis/painful bladder syndrome	No funding	16 (905)	8	Yes	Chonroitin sulfate	Global response assessment, pain, urinary frequency, urinary urgency, bladder capacity restoration	RCTs	F	Yes	Yes
Chung (2016); Hong Kong [92]	Chronic obstructive pulmonary disease	not reported	11 (925)	4	Yes	Chinese herbal medicine	Change in FEV1, St George’s Respiratory Questionnaire, 6-Minute Walk Test	RCTs	F	Yes	Yes
Dong (2016); Germany [96]	Lateral epicondylalgia	Not reported	27 (1913)	13	Yes	Peppering technique; prolotherapy	Change in pain scores	RCTs	B	Yes	Yes
Dulai (2016); USA [89]	Prevention of advanced metachronous neoplasia	No funding	15 (12,234)	10	Yes	Calcium, vitamin D, folic acid	Prevention of advanced metachronous neoplasia within 3-5 years of index colonoscopy, prevention of any metachronous neoplasia, risk of serious adverse events	RCTs	B	Yes	Yes
Howarth (2016); USA [104]	Exposure to domestic violence	Public	13 (1345)	11	Yes	Play therapy	child behavior disorders, child behavior symptoms, children’s mental health, depression, psychiatric symptoms, anxiety, self-harm, PTSD, school attendance or school functioning, children’s self-esteem, children’s happiness/social relationships, child quality of life, intervention of social services	RCTs	B	No	No
Huang (2016); China [74]	Acute promyelocytic leukemia	Public	21 (1666)	9	Yes	All-trans retinoic acid, realgar-Indigo naturalis formula	Event-free survival, complete remission, early death, remission time, hepatic toxicity, differentiation syndrome	RCTs	F	Yes	Yes
Linde (2016); Germany [108]	Depression	Public	100 (21,298)	22	Yes	St John’s Wort	Response to treatment (≥ 50% score reduction on a depression symptom severity scale)	RCTs	F	yes	no
Morrell (2016); UK [76]	Post-natal depression	Public	44 (NR)	6	Yes	Calcium, DHA, selenium	Maternal depression, anxiety, well-being	RCTs and SRs	B	No	Yes
Palmer (2016); Italy [109]	Chronic kidney disease	No funding	77 (12,562)	3	Yes	Calcium, iron	All-cause mortality, cardiovascular mortality, myocardial infarction, stroke, adverse events, serum phosphorus and calcium levels, coronary artery calcification	RCTs	F	Yes	Yes
Pompoli (2016); Italy [93]	Panic disorder	Public	54 (3021)	6	Yes	Psychodynamic therapies, physical therapy (e.g. breathing retraining, progressive muscle relaxation, applied relaxation)	Short-term remission of panic disorder (with or without agoraphobia), short-term response of panic disorder, dropouts for any reason	RCTs	B	Yes	Yes
Qin (2016); China [73]	Chronic prostatitis and chronic pelvic pain syndrome	Not reported	12 (1203)	7	Yes	Acupuncture, sham acupuncture, electroacupuncture	Change in total NIH-CPSI, changes in NIH-CPSI subscales, adverse events due to treatments	RCTs	B	Yes	Yes
Rochwerg (2016); Canada [94]	Idiopathic pulmonary fibrosis	No funding	19 (5694)	11	Yes	*N*-acetylcysteine (NAC)	Mortality, serious adverse events	RCTs	B	Yes	Yes
Sawangjit (2016); Malaysia [90]	Non-alcoholic fatty liver disease	Not reported	44 (3802)	11	Yes	Vitamin E and C	Fibrosis, death overall or related to liver and cardiovascular disease, cirrhosis, ballooning degeneration, steatosis, lobular inflammation, and NAS, mean changes in NAS, ballooning, steatosis, and lobular inflammation, adverse effects	RCTs	B	Yes	Yes
Skapinakis. P (2016); UK [75]	Obsessive-compulsive disorder	Public	54 (288)	17	Yes	St John’s Wort	Yale–Brown obsessive-compulsive scale (YBOCS)	RCTs	B	Yes	Yes
Wang (2016); China [91]	Rheumatoid arthritis	Public	22 (5255)	7	Yes	Tripterygium wilfordii Hook F	Treatment response (ACR 20, 50, or 70), patient evaluation of pain, blood acute-phase reactants, withdrawal of patients due to drug-emergent adverse events	RCTs	F	Yes	Yes
Wu (2016); Hong Kong [95]	Non-small cell lung cancer	Not reported	61 (4247)	12	Yes	Shen-qi-fu-zheng injection, Kang-ai injection, Compound ku-shen injection, Kang-la-te injection, Xiao-ai-ping injection, Zi-jin-long tablet, Shen-fu injection, Yi-fei-bai-du decoction, Fei-liu-ping extract, Hai-shen-su, extract from Tegillarca granosa, Fu-zheng-jiedu decoction	Quality of life	RCTs	F	No	Yes
Dong (2015); China [99]	Stroke prevention	Public	17 (86,393)	8	No	Folic acid, vitamin B6, vitamin B12, niacin	Risk of stroke, cerebral infarction, and cerebral hemorrhage	RCTs	B	Yes	No
Dong (2015); China and Germany [43]	Shoulder impingement syndrome	No funding	33 (2300)	4	Yes	Acupuncture, kinesio taping therapy, pulsed electromagnetic field therapy	Pain score	RCTs	B	Yes	Yes
Gartlehner (2015); USA [60]	Major depressive disorder	Public	127 (NR)	10	Yes	Omega-3 fatty acids, Acupuncture, S-adenosyl methionine, St. John’s wort	Response to treatment, remission, speed of response, speed of remission, relapse, quality of life, functional capacity, reduction of suicidal ideas or behaviors, reduction of hospitalization, overall adverse events, withdrawals due to adverse events, serious adverse events, specific adverse events	RCTs	F	No	No
Grant (2015); USA [48]	Menopausal symptoms	Public	283 (NR)	8	Yes	Black cohosh, ginseng, isoflavones	Vasomotor symptoms, quality of life, psychological, sexual function, sleep disturbance	RCTs	B	Yes	Yes
Kongtharvonskul (2015); Thailand [101]	Osteoarthritis (knee)	No funding	31 (NR)	4	Yes	Glucosamine	Pain, total and subWOMAC scores (pain, stiffness, and function), Lequesne algofunctional index, joint space width, adverse events	RCTs	F	No	No
Lehert (2015); USA [98]	Cognitive aging	Public	24 (NR)	11	Yes	B-Vitamins, Tai Chi, Vitamin D, Yoga, Omega-3, soy isoflavones	Global cognition, episodic memory	RCTs	F	Unclear	No
Lewis (2015); UK [38]	Sciatica	Public	122 (NR)	21	Yes	Manipulation, acupuncture, passive physical therapy, radiofrequency treatment	Global effect, pain intensity	RCTs; NRS	B	No	Yes
Linde (2015); Germany [39]	Depressive disorders	Public	66 (15,161)	9	Yes	St. John's Wort	Efficacy; discontinuation due to adverse effects	RCTs	B	Yes	No
Loveman (2015); UK [100]	Idiopathic pulmonary fibrosis	Public	11 (3294)	6	Yes	*N*-acetylcysteine (NAC, Triple NAC, and inhaled NAC)	Decline in forced vital capacity	RCTs	B	Yes	No
Reinecke (2015); Germany [110]	Chronic pain	Public	46 (10,742)	5	Yes	Physiotherapy (hydrotherapy, osteopathic intervention vs. sham, active non-invasive interactive neurostimulation, balneotherapy, Qigong, transcutaneous electrical nerve stimulation, reflexology, electromagnetic field therapy, hypnosis	Pain, analgesic effects, adverse events	RCTs	F	No	No
Steenhuis (2015); Netherlands [97]	Psychotic disorders	Public	10 (576)	5	Yes	Music therapy, yoga therapy	Depressive symptoms	RCTs	F	Yes	No
Zeng (2015); China [40]	Osteoarthritis (knee)	Public	20 (995)	7	no	Transcutaneous electrical nerve stimulation, neuromuscular electrical stimulation, interferential current, pulsed electrical stimulation, non-invasive interactive neurostimulation	Pain intensity, change in pain score	RCTs	B	Yes	Yes
Zhu (2015); China [41]	Hepatic encephalopathy	Public	20 (1007)	6	Yes	L-ornithine-L-aspartate, branched chain amino acids	Clinical improvement, blood ammonia concentration, mental status, adverse effects	RCTs	B	Yes	Yes
Gerger (2014); Switzerland [35]	Post-traumatic stress disorder (PTSD)	Public	66 (4190)	8	No	EMDR and stress management (includes some forms of relaxation and biofeedback)	Severity of PTSD symptoms	RCTs	B	Yes	No
Griebeler (2014); USA [36]	Diabetic neuropathy	Public	65 (12,632)	10	Yes	Topical capsaicin	Pain relief	RCTs	B	Yes	No
Kriston (2014); Germany [34]	Persistent depressive disorder	Public	45 (11,154)	9	Yes	Acetyl-l carnitine	Treatment response (≥ 50% improvement), acceptability	RCTs	B	Yes	No
Wang (2014); China [37]	Gastric cancer	Not reported	38 (2761)	10	No	Chinese Herb Injections: Aidi injection, Astragalus polysaccharides injection, Cinobufacini injection, Compound matrine injection, Delisheng injection, Ginseng polysugar injection, Kangai injection, Kanglaite injection, Shenqifuzheng injection, Yadanziyouru injection	Karnofsky (KPS) score, overall response rate, nausea, and vomiting, leukopenia	RCTs	B	No	No
Cawston (2013); Germany [32]	Chronic low back pain	Industry	15 (5374)	18	Yes	Glucosamine	Treatment efficacy	RCTs	B	No	No
Corbett (2013); UK [114]	Osteoarthritis (knee)	Public	114 (9709)	9	Yes	Acupuncture, balneotherapy, neuromuscular electrical stimulation, pulsed electrical stimulation, pulsed electromagnetic fields, static magnets, Tai Chi, TENS	Pain	RCTs	B	Yes	Yes
Nüesch (2013); Switzerland [31]	Fibromyalgia syndrome	Public	102 (14,982)	11	Yes	Balneotherapy	Pain, quality of life	RCTs	B	Yes	Yes
Snedecor (2013); USA [33]	Painful diabetic peripheral neuropathy	Industry	58 (11,883)	32	Yes	Capsaicin, alpha-lipoic acid, sativex	Pain reduction	RCTs	B	Yes	Yes
Thakkinstian (2012); Unclear [30]	Chronic prostatitis and chronic pelvic pain syndrome	Not reported	19 (1669)	5	Yes	Phytotherapy (not specified)	Total symptom scores, pain scores, voiding score, quality of life, treatment response	RCTs	F	No	No
Anothaisintawee (2011); Thailand [29]	Chronic prostatitis and chronic pelvic pain syndrome	Public	23 (2315)	8	Yes	Phytotherapy (not specified)	Total symptom scores, pain score, voiding score, QoL score,	RCTs	F	No	No
Imamura (2010); UK [27]	Stress urinary incontinence	Public	55 (6608)	14	Yes	Electrical stimulation	Cure rate, improvement rate	RCTs and quasi-RCTs	B	No	Yes
Wandel (2010); Switzerland [28]	Osteoarthritis (hip, knee)	Public	10 (3803)	4	No	Chondroitin and glucosamine	Pain	RCTs	B	Yes	No

Abbreviations: *ADE* adverse drug event, *ADR* adverse drug reaction, *IFC* interferential current, *NIN* non-invasive interactive neurostimulation, *NMES* neuromuscular electrical stimulation, *NRS* non-randomized study, *PEMF* pulsed electromagnetic fields, *PES* pulsed electrical stimulation, *QoL* quality of life, *TENS* transcutaneous electrical nerve stimulation

*where a review included multiple analyses of varying size, the minimum number of interventions compared, studies included and patients included is provided

**Fig. 2:**
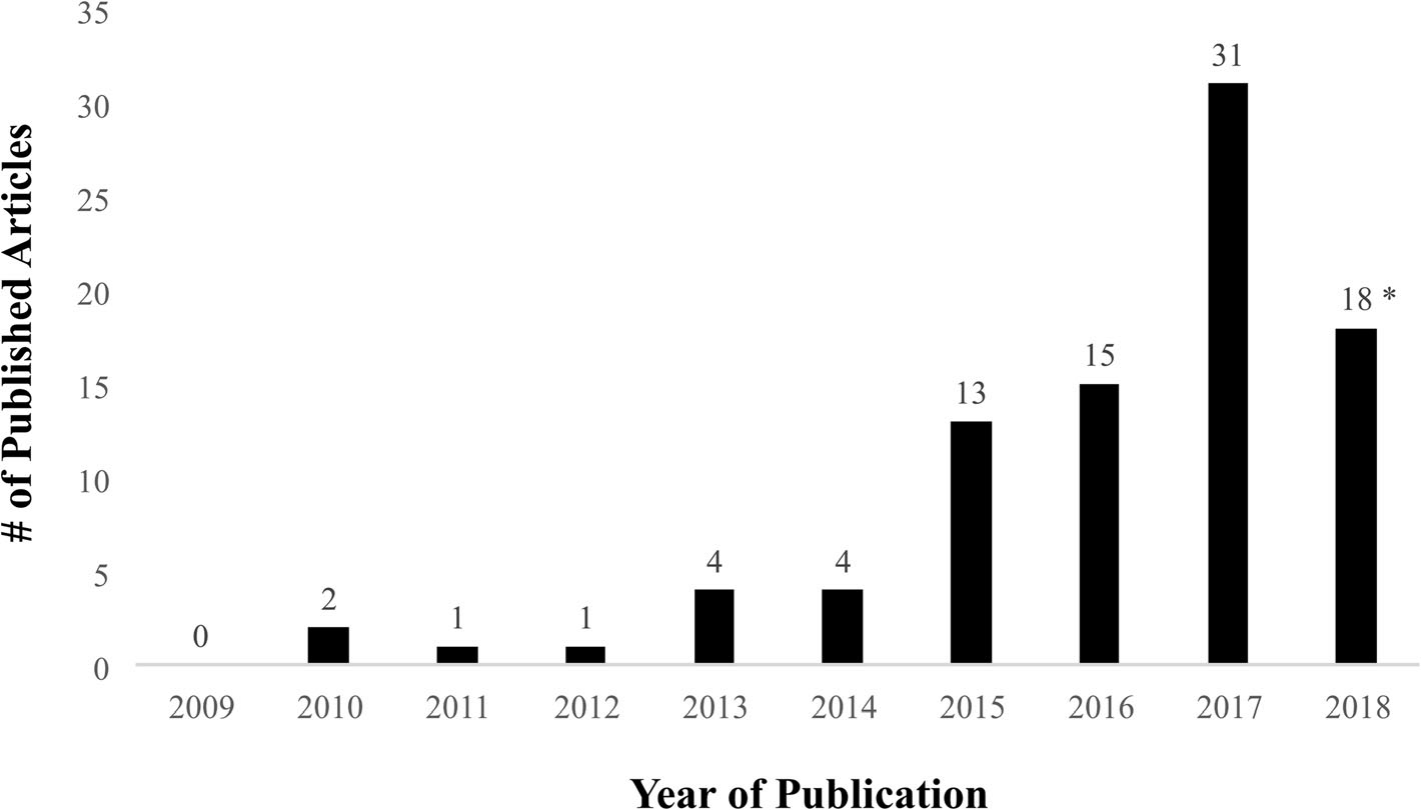
Number of published NMAs with CAM interventions per year. The distribution of publication year of the set of included reviews is shown. “*” denotes that the search was performed in May 2018, and thus only 5 months of that year are reflected in this review

**Fig. 3:**
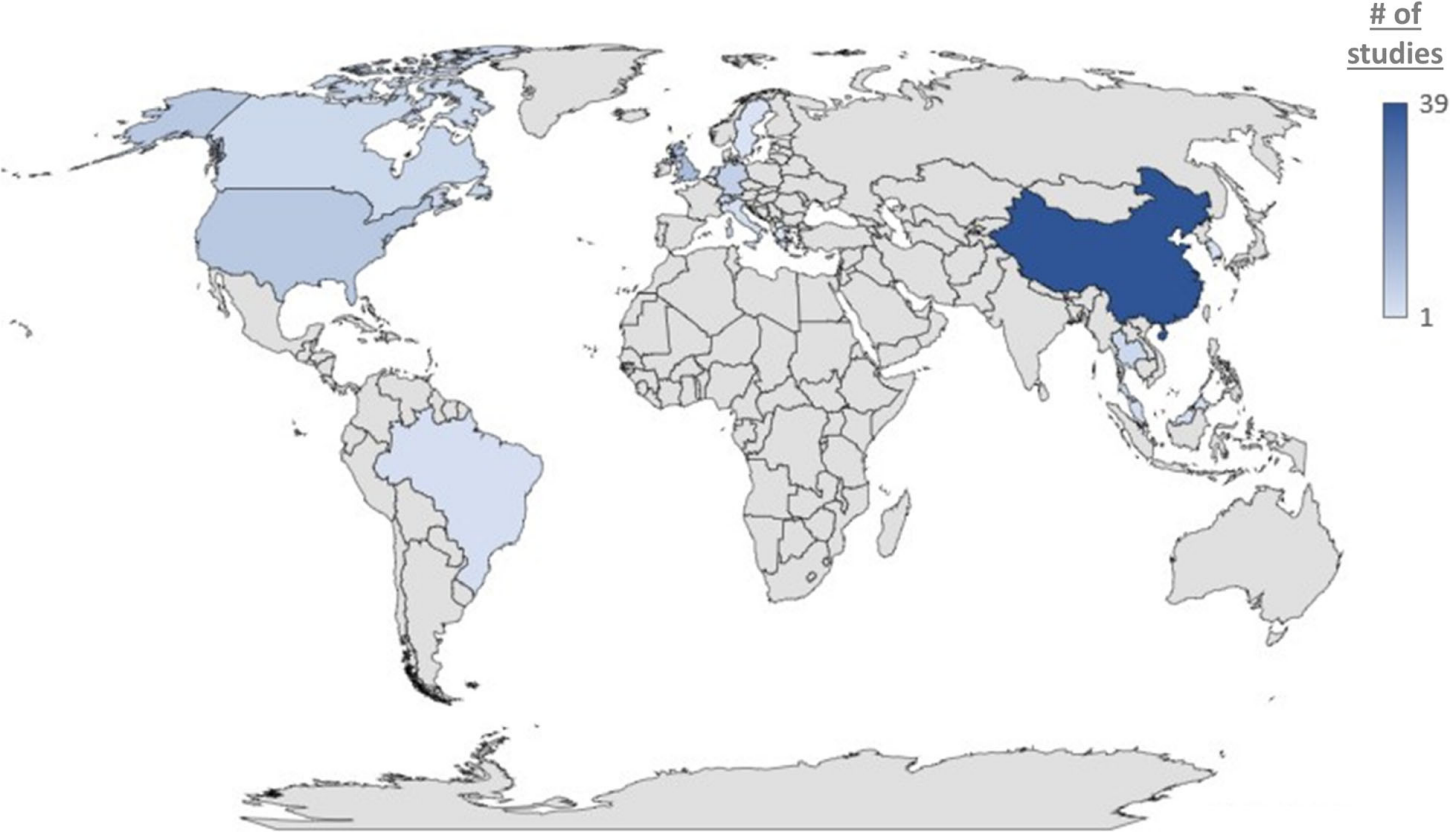
Countries producing published NMAs involving CAM interventions. A heat map is presented displaying the number of NMA publications produced by different countries (according to the first authors’ affiliation). Darker shades of blue denote countries that have produced larger numbers of studies

Year of publication amongst the included reviews ranged from 2010 to 2018 (median 2017; Fig. 2). A total of 35 (39%) were conducted in China, 11 (12.4%) were conducted in the United Kingdom, 8 (9.0%) were conducted in the United States, and 6 (6.7%) were conducted in Germany; 4 (4.5% per country) were conducted in each of Canada, Switzerland, and Thailand, 3 (3.4%) were conducted in each of the Netherlands and Hong Kong, 2 were conducted in each of Italy (2.3%), Malaysia (2.3%), and Brazil (2.3%), and single reviews were conducted in Korea, Sweden, and Greece (see Table 1); Fig. 3 presents a heat map summarizing the distribution of nations producing the set of included NMAs. Funding was public for 57 reviews (64.0%), private/industry sponsored for 3 reviews (3.3%), and no funding was available for support for 12 reviews (13.5%) (see Table 1); funding was unreported for 17 reviews (19.1%).

### Patient indications and outcomes studied

Table 2 provides a listing of the patient indications that were studied within the included reviews, as well as data regarding both the totality of reviews per indication and the evolution of reviews with CAM interventions between 2010 and 2018. A total of 10 were from the realm of mental health, addressing topics such as depressive disorder, post-traumatic stress disorder, post-natal depression, treatment-resistant depression, obsessive-compulsive disorder, psychotic disorders, panic disorder, and attention deficit hyperactivity disorder [34, 35, 39, 45, 60, 75, 76, 80, 93, 108]. A total of 11 reviews related to cancer were identified, including NMAs of interventions for gastrointestinal cancer, pancreatic cancer, acute promyelocytic leukemia non-small cell lung cancer, neurotoxicity from chemotherapy, and cancer-related fatigue [37, 54, 66, 74, 82, 89, 95, 105, 107, 112, 113]. Osteoarthritis (including prostatitis) was the subject of 10 reviews [28–30, 40, 42, 73, 78, 101, 103, 114], gastrointestinal infections/disorders were the subject of 6 reviews [61, 64, 65, 67, 68, 115], cardiovascular disease in 4 reviews [46, 69, 99, 102], topics related to pregnancy, childbirth and newborn health in 4 reviews [47, 62, 71, 83], and a variety of other clinical indications were assessed in 3 or fewer reviews. The number of NMAs overall increased notably from earlier to later years.

**Table 2:** Time horizon of NMA publications by clinical indication

Condition studied	Distribution of reports by year of publication									
	2010	2011	2012	2013	2014	2015	2016	2017	2018	Total
Acute promyelocytic leukemia							1			1
Attention deficit hyperactivity disorder								1		1
Alzheimer’s disease								1	1	2
Antibiotic-associated diarrhea								1		1
Atrophic vaginitis									1	1
Blood pressure reduction								1		1
Bulemia nervosa									1	1
Cancer-related fatigue									1	1
Cerebral infarction									1	1
Chronic ankle instability									1	1
Chronic fatigue syndrome								1		1
Chronic kidney disease							1			1
Chronic kidney disease mineral and bone disorder								1		1
Chronic low back pain				1						1
Chronic obstructive pulmonary disease							1			1
Chronic oro-facial pain								1		1
Chronic pain						1				1
Chronic prostatitis and chronic pelvic pain syndrome		1	1				1			3
Cognitive aging						1				1
Crohn's disease recurrence								1		1
Depression							1			1
Depressive disorders						1				1
Diabetic neuropathy					1			1		2
Exposure to domestic violence							1			1
Fibromyalgia syndrome (FMS)				1						1
Functional dyspepsia								1		1
Gastric cancer					1				1	2
Gastrointestinal cancer								2		2
Helicobacter pylori infection								3		3
Hepatic encephalopathy						1				1
Idiopathic pulmonary fibrosis						1	1			2
Infantile rotavirus enteritis			1							1
Insomnia									1	1
Interstitial cystitis/painful bladder syndrome								1		1
Kashin-Beck disease									1	1
Lateral epicondylalgia							1			1
Macular degeneration									1	1
Major depressive disorder						1				1
Menopausal symptoms						1				1
Myofacial pain syndrome								1		1
Necrotizing enterocolitis								1		1
Neurotoxicity from chemotherapy								1		1
Non-alcoholic fatty liver disease							1			1
Non-small cell lung cancer							1			1
Obsessive-compulsive disorder							1			1
Osteoarthritis (hip or knee)	1			1		2		2	1	7
Painful diabetic peripheral neuropathy				1						1
Pancreatic cancer								1		1
Panic disorder							1			1
Persistent depressive disorder					1					1
Post-natal depression							1			1
Post-operative complications								1		1
Post-stroke recovery								1		1
Preeclampsia and gestational hypertension								1		1
Pressure ulcers								1		1
Pre-term birth measures*									1	1
Preventing oxaliplatin-induced peripheral neurotoxicity								1		1
Prevention of advanced metachronous neoplasia							1			1
Prevention of respiratory tract infection								1		1
Prevention of type 2 diabetes in patients with prediabetes									1	1
Psoriasis								1		1
Psychologic preparation for surgery under general anesthesia									1	1
Psychotic disorders						1			1	2
Post-traumatic stress disorder					1					1
Rheumatoid arthritis							1			1
Sciatica						1				1
Shoulder impingement syndrome						1				1
Spasticity in multiple sclerosis									1	1
Stable angina									1	1
Stimulation of breast milk production									1	1
Stress urinary incontinence	1									1
Stroke prevention						1				1
Vasomotor symptoms								1		1

A display of both year-by-year evolution of CAM-related NMAs by indication as well as the total number of NMAs per indication is provided. Numbers reported within individual cells refer to the # of NMAs in a calendar year.

*mortality, necrotizing enterocolitis, late-onset sepsis, time to full enteral feed

The outcomes studied within each review were also collected. While a narrative overview of these endpoints is not provided here given the extensive nature of this information, a detailed listing for each review has been included in Table 1.

### Interventions reviewed and network geometry

**Fig. 4:**
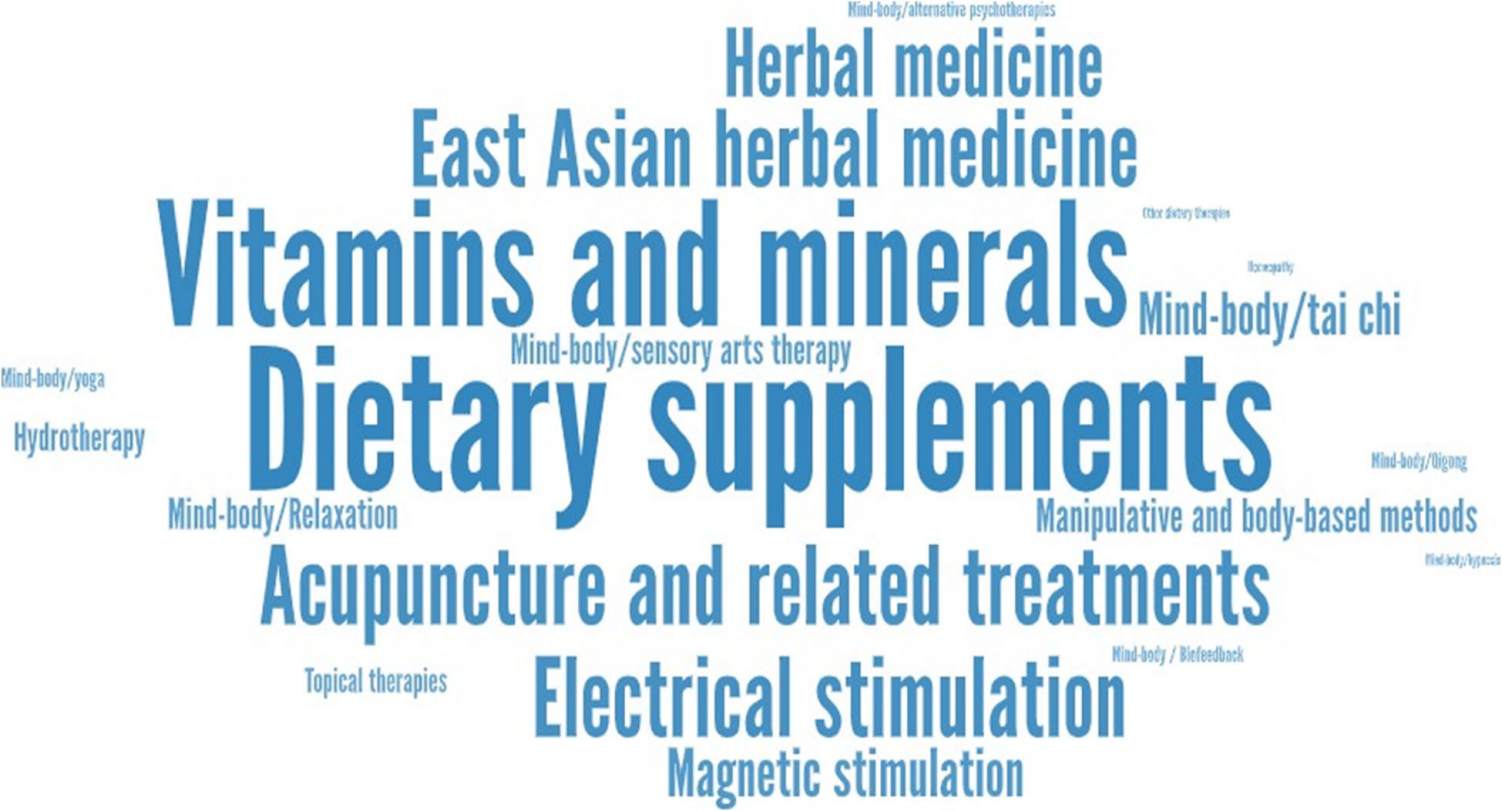
World cloud displaying the frequency of NMAs involving forms of CAM therapy. A word cloud presenting the frequencies of inclusion of different treatments in the set of included reviews is shown. Larger font denotes interventions which appeared more frequently. The most common interventions were dietary supplements and herbal medicines

A total of 51 reviews (56.7%) considered more than one form of CAM intervention (median 2; IQR 1–4; range 1–18). A total of 17 reviews (19.1%) involved comparisons between CAM interventions only [28, 35, 37, 40, 43, 46, 47, 55, 61, 62, 65, 68, 71, 87, 102, 106, 116], while the remaining 72 (80.9%) also involved comparisons with general medical interventions (Table 1). Figure 4 presents a word cloud summarizing the types of CAM interventions that were identified within the included set of review articles. Dietary supplements (*n* = 42) and vitamins and minerals (*n* = 35) appeared in the largest number of reviews, followed by acupuncture and related treatments (*n* = 20), electrical stimulation (*n* = 20), East Asian herbal medicines (*n* = 19), herbal medicines (*n* = 18), and magnetic stimulation (*n* = 10); all other interventions were assessed in fewer than 10 reviews. The total number of nodes per evidence network (both CAM and non-CAM interventions) ranged from 3 to 32 (median 8). The total number of patients ranged from 288 to 86,393 (median 3146; IQR 1710 to 8488) for the 82 reviews where this information was available; the numbers of studies ranged from 5 to 283 (median 27; IQR 20 to 55).

### Statistical methods and completeness of reporting

Amongst the included reviews, 60 (66.7%) performed analyses using a Bayesian model for NMA while the remaining 29 (32.2%) used a frequentist approach (see Table 1). Consideration of the appropriateness of the consistency assumption was discussed in 70 (78.7%) reports. In addition to reporting of primary findings using approaches such as tables, forest plots, and league tables, a total of 63 (70.8%) NMAs reported either values of Surface Under the Cumulative Ranking (SUCRA) curve, rank-o-grams of probabilities, the probability of being best for each treatment or an average/median ranking per intervention in terms of secondary measures of summary effect (see Table 1).

**Fig. 5:**
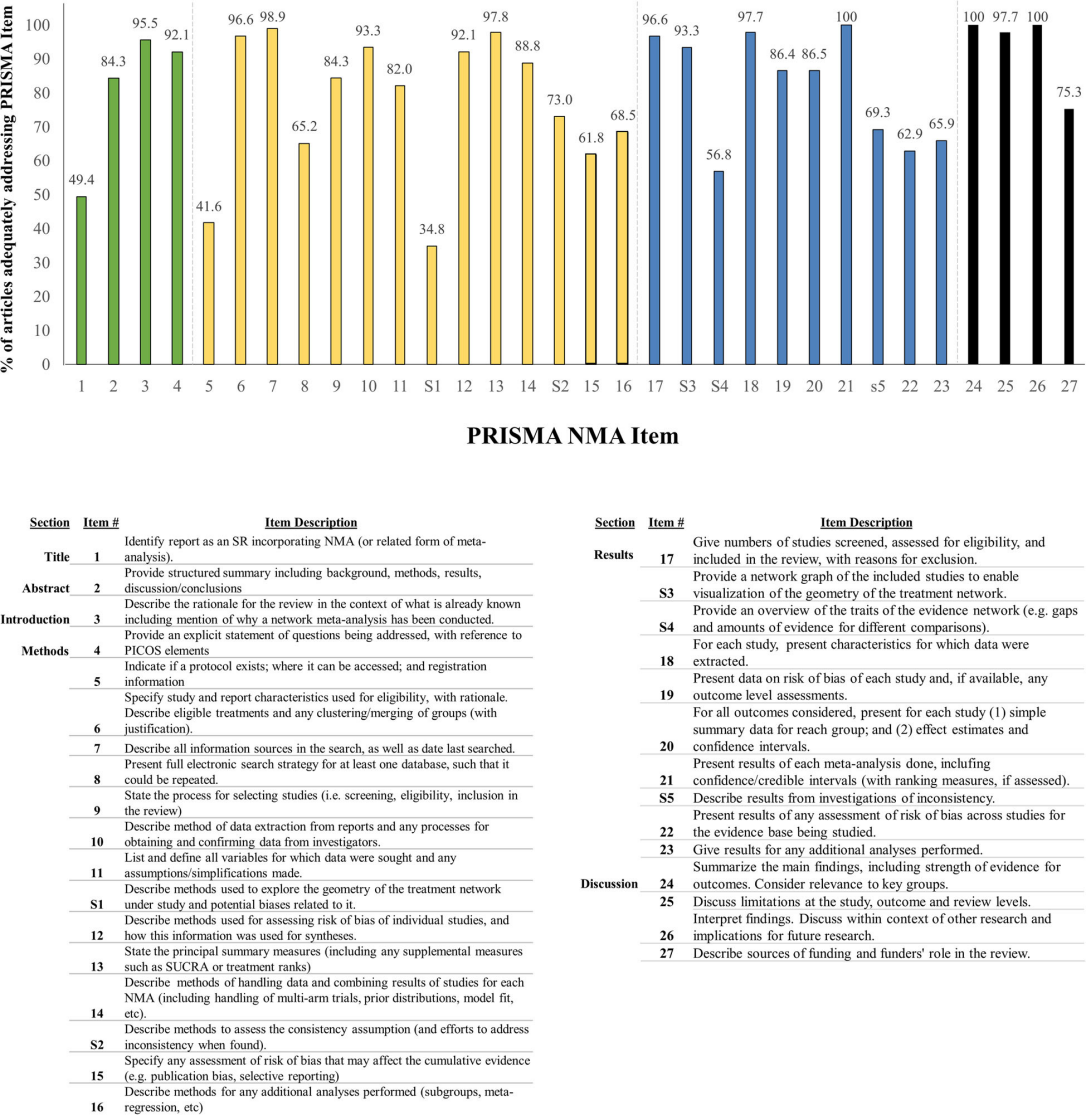
Graded completeness and transparency of reporting (PRISMA-NMA; *n* = 89 reviews). A bar chart presenting the judged compliance of included reviews with recommendations from the PRISMA extension statement for NMA is shown, stratified by component of the guidance. The supplemental items (S1–S5) specific to NMA are bolded and underlined on the horizontal axis

With regard to the completeness of reporting, the proportion of included NMAs adequately addressing each of the 32 items from the PRISMA NMA Checklist is summarized in Fig. 5 (an overview of the PRISMA NMA Checklist is provided in Additional file 3, while Additional file 4 contains a detailed account of the study-specific assessments). For twenty checklist items (but only one of the 5 added checklist items specific to NMA), reporting was judged to be adequate for 80% or more of the reviews assessed; this included core elements of the abstract, introduction, and methods (specification of eligibility criteria, search information sources, process for study selection, methods for data collection, variables extracted, risk of bias appraisal methods, principal summary measures, methods for meta-analysis), as well as certain components of the findings and discussion sections (numeric details of study selection, provision of a network graph, presentation of study characteristics, presentation of risk of bias data, summary data related to included studies, appraisal of the risk of bias across studies, a summative overview of findings, discussion of study limitations and interpretations).

Several other checklist items were associated with less common completeness of reporting. Amongst the 89 included reviews, only 44 (49.4%) identified the report as a systematic review incorporating a NMA (Checklist Item 1). Few studies adequately reported whether a review protocol existed, and where to access the protocol (Checklist Item 5; 37/89 or 41.6%). A full electronic search strategy for at least one database was provided by only 58 of 89 included studies (65.2%; Checklist Item 8), while totals of 55 (61.8%) and 61 (68.5%) studies addressed methodologic details related to the risk of bias assessments across studies (e.g., publication bias, Checklist Item 15) and details of additional analyses (Checklist Item 16); regarding the latter two elements, reporting was also less complete within the results of the included reviews (Checklist Items 22 and 23). Funding and funder roles were also inconsistently reported (Checklist Item 27). With regard to Checklist Items S1–S5 that are specific to NMA, only one exceeded 80% adequate reporting across the included reviews (Checklist Item S3—provision of a network graph). Methods used to explore network geometry (Checklist Item S1), methods to assess for inconsistency of direct and indirect evidence (Checklist Item S2), description of the traits of the evidence network (Checklist Item S4) and findings from analyses checking for inconsistency (Checklist Item S5) were adequately reported in totals of 34.8%, 73.0%, 56.8%, and 69.3%, respectively.

In reviewing the distribution of the median (IQR) total number of PRISMA items reported over time, findings suggest that the reporting transparency of network meta-analyses has improved slightly over time in NMAs with CAM interventions. In 22 included reviews published prior to 2016 (date chosen in relation to the publication of the PRISMA extension statement for NMA in June 2015), the median (interquartile range) number of items addressed out of 32 (i.e., 27 core items and 5 NMA-related items) was 25 (IQR 23-27.5). In the set of 67 reviews published since the start of 2016, the corresponding median was 26 (IQR 24-28). Totals of 41 (61.2%) reviews published in 2016 and afterward adequately addressed 25 or more checklist items, while the corresponding total amongst those published in 2015 and earlier was 7 (31.8%). With regard to NMA-specific reporting items (S1–S5), improvements were noted in the more recent category of publications for S2 regarding inconsistency methods (79.7% versus 56.0%), S3 regarding provision of network diagrams (96.9% versus and 84.0%) and S5 regarding findings from inconsistency evaluations (70.3% versus 64.0%), while the proportions of studies for S1 and S4 regarding assessment of network geometry patterns were similar across time periods.

## Discussion

The growth of NMA as an incrementally important knowledge synthesis methodology for the comparison of healthcare interventions is well established [16]. While its value in informing the comparison of multiple pharmacologic therapies, in particular, is well known, the use of NMA in evaluating the benefits of CAM interventions, to our awareness, has not previously been studied. In the current scoping review, we have enhanced the current understanding of its history of use in the CAM realm.

Several interesting findings were identified in the context of this scoping review. First, the annual frequency of NMAs incorporating one or more CAM interventions has risen considerably since 2010, aligning with the type of relative growth observed with NMAs in general. While the largest number of reviews included in this study was produced in China, the diverse range of countries represented was geographically diverse, corroborating the use of NMA to be global in nature. The range of CAM interventions studied and the assortment of medical diagnoses in which they were assessed were also diverse, with certain most common approaches to treatment (including dietary supplements, vitamins, minerals, and East Asian herbal medicines) being observed. From a design perspective, the current review suggests that in many cases, CAM interventions were considered either in separation from conventional medicine (compared only with other CAM therapies) or only a very limited amount of CAM therapies were included in comparisons with conventional medicine. The rationale for both occurrences may potentially be driven by the uncertainty of many researchers as to the benefits that CAM interventions as a whole may potentially offer patients; other plausible rationale may include the stages of disease assessed in reviews (CAM therapies may be tried earlier or later in different cases), the types of benefits that are sought by physicians and patients (e.g., symptom relief versus the impact on disease progression), or concerns regarding potential differences in patient populations (i.e., the potential for systematic differences between those agreeing to receive CAM versus non-CAM interventions). Strategies to enhance their inclusion may, therefore, require greater collaboration amongst CAM experts and producers of systematic reviews to establish more diverse research teams, in particular at the design phase of systematic reviews, to grow the list of comparators for consideration; however, this may not address all existing challenges.

The collection of systematic reviews incorporating NMAs identified in this scoping review offers opportunities in several directions. From the perspective of planning future research, the listing of included reviews may allow organizations with a focus in CAM interventions to (a) identify clinical diagnoses considered highly amenable to CAM therapies where no prior NMA has been conducted, allowing for plans to address a current knowledge gap; (b) identify reviews for high priority indications where a comprehensive comparison amongst CAM therapies has not yet been conducted, with past reviews focused upon only a very limited selection; (c) identify reviews for high priority indications where there remains a clear need to derive treatment comparisons between CAM and conventional medicines; and (d) to consider possible conditions wherein future randomized trials of CAM therapies may be informative. While not discussed in detail in the text of this review, the summary table of past reviews also lists the considered outcomes from past NMAs for consideration by multiple audiences to allow thought as to ways existing information might be helpful or to enhance plans for future research in syntheses related to clinical areas assessed in prior reviews. Surveys indicate that the most commonly used CAM therapies in the US are non-vitamin, non-mineral dietary therapies [117], and this is consistent with the relative prominence of dietary supplements observed in this scoping review. The next most commonly used CAM therapies are deep breathing exercises, yoga, chiropractic or osteopathic manipulation and meditation, and more recent US research also indicates that the percentage of persons using yoga, meditation or chiropractic therapies is increasing [117]. These therapies appeared less often in NMAs, and with increased use, these therapies may be a focus of future research comparisons. CAM therapies are used by a large proportion of people diagnosed with chronic conditions [118], particularly musculoskeletal pain conditions such as arthritis [119]. Although many people who use CAM do so for musculoskeletal pain or mental health [120], many people with musculoskeletal pain conditions who use CAM do not use the CAM to treat pain [121]. Likewise, some of the most commonly used CAM modalities such as dietary supplements or yoga are most frequently used for “wellness” reasons rather than treatment of a condition [122]. Identifying where appropriate CAM therapies could be incorporated into NMAs, therefore, cannot rely only upon the prevalence of use, but rather will also consult with researchers and clinicians to identify gaps in the NMA literature. This scoping review may assist in this identification.

In reviewing the completeness and transparency of reporting of the set of included NMAs, several weaknesses were identified relative to both core elements of PRISMA as well as certain elements specific to the PRISMA Extension statement for NMA; this aligns with past evaluations of published NMAs [123], and efforts to enhance both elements are needed. From a methodologic perspective, further research considering specific elements that relate to the conduct and assumptions underlying NMA may also be relevant. For example, the appropriateness of “lumping” control groups (such as different forms of sham therapy, placebo, and waitlist controls) requires consideration and has been shown previously to potentially introduce bias into the findings of NMAs based upon differential event rates or mean values between sources of control [124–127]. Furthermore, careful consideration as to whether the study populations enrolled in trials of CAM interventions may differ in important ways relative to those enrolled in trials of conventional methods may also present challenges to the transitivity assumption. In our analyses that looked at trends in reporting completeness based upon PRISMA NMA over time, the median (and IQR) numbers of elements addressed were similar before and after 2016, though the proportions of studies before and after this date that addressed totals of > 25 items (61.2% versus 31.8%) and > 30 items (4.5% versus 0%) both were improved in the latter group.

There are certain limitations to this review to be noted. First, while this scoping review set out to map the conditions studied, CAM interventions evaluated, reporting completeness and other elements, judgements as to the appropriateness of methods for NMA and the completeness of interventions compared in NMAs (from a clinical relevance perspective) were not drawn; while of interest, these were considered to be beyond the goals for this research. Second, while certain characteristics of the NMAs were associated with failures to provide related information within the article text, we did not contact authors for these details, instead, we rely upon what was described only in the article. Last, we did not search registration records for ongoing systematic reviews that may be oriented toward the comparison of CAM therapies or involve comparisons between CAM and conventional medical interventions, and thus the data presented here may underestimate the extent of ongoing NMA evaluations involving CAM therapies.

## Conclusion

The application of NMA methods to inform comparisons of CAM interventions has grown rapidly in recent years, and the diversity of interventions assessed and conditions studied is diverse. Given the prevalence of use of CAM interventions, particularly for musculoskeletal conditions and mental health, future efforts to incorporate comparisons in NMAs with conventional medicines and to identify and address the methodologic challenges of NMA in this setting are worthwhile for the comprehensive identification and comparison of treatment options. This review may serve as a starting point from which future research initiatives related to the evaluation of CAM interventions can be prioritized.

A completed PRISMA for Scoping Reviews Checklist is provided in Additional file 5 to document the completeness of reporting of this review.

## Supplementary information

**Additional file 1.** Literature Search Strategies for the review are provided

**Additional file 2.** The list of eligible CAM Interventions is provided

**Additional file 3.** A copy of the PRISMA NMA Extension Checklist is provided for reference

**Additional file 4.** The completed PRISMA-NMA Assessments for the included studies are provided

**Additional file 5.** A completed PRISMA for Scoping Reviews Checklist for the current review is provided

## Data Availability

All data generated or analyzed during this study are included in this published article [and its supplementary information files].

## Abbreviations

CAM: Complementary and alternative medicine
NMA: Network meta-analysis
PRISMA: Preferred Reporting Items for Systematic Reviews and Meta-Analyses
RCT: Randomized controlled trial
SR: Systematic review
SUCRA: Surface under the cumulative ranking
