# Supplementary material for: A scoping review of network meta-analyses assessing the efficacy and safety of complementary and alternative medicine interventions
Source: Syst Rev. 2020 Apr 30;9:97. doi: 10.1186/s13643-020-01328-3 (PMC7191816; doi:10.1186/s13643-020-01328-3)
Supplement: Supplementary file 1 — Additional file 1. Literature Search Strategies for the review are provided [file 13643_2020_1328_MOESM1_ESM.docx]

**Additional File 1: Literature Search Strategies**

**Database:** Ovid MEDLINE(R) In-Process & Other Non-Indexed Citations and Ovid MEDLINE(R)

--------------------------------------------------------------------------------

1     (network* adj3 (meta-analy* or metanaly* or metaanaly* or met analy*)).mp.

2     (MTC adj3 (meta-analy* or metanaly* or metaanaly* or met analy*)).mp.

3     (indirect* adj comparison*).mp.

4     (mixed treatment* adj comparison*).mp.

5     (multiple treatment* adj comparison*).mp.

6     (multi-treatment* adj comparison*).mp.

7     (simultaneous* adj comparison*).mp.

8     mixed comparison*.mp. (10)

9     (umbrella adj3 (meta-analy* or metanaly* or metaanaly* or met analy*)).mp.

10     or/1-9

**Database:** Embase Classic+Embase

--------------------------------------------------------------------------------

1 (network* adj3 (meta-analy* or metanaly* or metaanaly* or met analy*)).mp.

2 (MTC adj3 (meta-analy* or metanaly* or metaanaly* or met analy*)).mp.

3 (indirect* adj comparison*).mp.

4 (mixed treatment* adj comparison*).mp.

5 (multiple treatment* adj comparison*).mp.

6 (multi-treatment* adj comparison*).mp.

7 (simultaneous* adj comparison*).mp.

8 mixed comparison*.mp.

9 (umbrella adj3 (meta-analy* or metanaly* or metaanaly* or met analy*)).mp.

10 or/1-9

**Database:** Cochrane

1 (network* near/3 ((meta next analy*) or metanaly* or metaanaly* or (met next analy*)))

2 (MTC near/3 ((meta next analy*) or metanaly* or metaanaly* or (met next analy*)))

3 (indirect next comparison*)

4 (mixed next treatment* next comparison*)

5 (multiple next treatment* next comparison*)

6 (multi next treatment* next comparison*)

7 (simultaneous* next comparison*)

8 (mixed next comparison*)

9 (umbrella near/3 ((meta next analy*) or metanaly* or metaanaly* or (met next analy*)))

10 {or #1-#9}
