# Supplementary material for: A scoping review of network meta-analyses assessing the efficacy and safety of complementary and alternative medicine interventions
Source: Syst Rev. 2020 Apr 30;9:97. doi: 10.1186/s13643-020-01328-3 (PMC7191816; doi:10.1186/s13643-020-01328-3)
Supplement: Supplementary file 2 — Additional file 2. The list of eligible CAM Interventions is provided [file 13643_2020_1328_MOESM2_ESM.docx]

**Additional File 2: CAM Interventions List**

The table below provides a comprehensive list of CAM interventions that would be of relevance to the work being carried out in collaboration with the Cochrane Complementary Medicine Field. While the list is not exhaustive, it represents a vast majority of available CAM therapies. Note that while the list attempts to be inclusive and there are many therapies listed that not all audiences would consider to be CAM, the Cochrane Complementary Medicine Field explicitly considers many vitamin or mineral therapies (eg, addressing vitamin deficiencies or delivered intravenously in hospital settings) and most exercise and conventional psychological therapies to be outside of CAM (cam.cochrane.org/ operational-definition-complementary-medicine). For the work outlined above, network meta-analyses involving any of the therapies listed below are of interest. This includes both NMAs comparing only CAM therapies and NMAs comparing CAM therapies with other more conventional healthcare interventions.

| **CAM interventions, A-D** | **CAM interventions, E-K** | **CAM interventions, L-R** | **CAM interventions, S-Z** |
| --- | --- | --- | --- |
| Açaí / Euterpe oleracea | Echinacea | Laetrile | S-Adenosyl methionine (SAM-e) |
| Acupressure | EDTA (ethylenediaminetetraacetic acid) when used in chelation therapy as described above (see Chelation therapy) | Laser acupuncture | Safflower Yellow injection (a Chinese herbal medicine) |
| Acupuncture | Eicosapentaenoic acid (EPA) (an omega-3 fatty acid) supplements | Laughter therapy | Salacia oblonga |
| Acustimulation / acupoint stimulation | Electric stimulation therapy | Lentinan (derived from Shitake) | Salvia (miltiorrhiza)(injection) (a Chinese herbal medicine) |
| African prune / Prunus Africana / Pygeum africanum) | Electroacupuncture | Light therapy / phototherapy (exclude for treatment of seasonal affective disorder, eczema, psoriasis, neonatal jaundice) | Sanchi preparations (a Chinese herbal medicine) |
| Aiyishu (a Chinese herbal medicine) | Electroacupuncture according to Voll (a diagnostic method) | Linoleic acid (an omega-6 fatty acid) supplements | Saw palmetto / serenoa repens |
| Alexander technique | Electromagnetic stimulation therapy | L-isoleucine (an amino acid) supplements | Selenium supplements |
| Alpha-linolenic acid (ALA) (an omega-3 fatty acid) supplements | Electromagnetic therapy (exclude electromagnetic field therapy for delayed- and non-union fractures) | Liuwei dihuang pill (a Chinese herbal medicine) | Shamanistic medicine / Shamanism |
| Amino acid supplements | Electrotherapy | L-leucine (an amino acid) supplements | Shark cartilage |
| Angelica | Elemental diet | Low fat diets | Shengmai / shenmai (a Chinese herbal medicine) |
| Anthroposophic medicine | Energy field work | Low protein diets | Shenqi Fuzheng (a Chinese herbal medicine) |
| Antioxidant supplements | Essiac formula | Low-glycemic index diets | Shensu / shenfu (a Chinese herbal medicine) |
| Arachidonic acid (AA or ARA) (an omega-6 fatty acid) supplements | Estrogen (exclude for treatment of natural or surgical menopause) supplements | L-threonine (an amino acid) supplements | Shexiang (injection) (a traditional Chinese medicine] |
| Aromatherapy | Evening primrose oil | L-valine (an amino acid) supplements | Shiatsu |
| Art therapy | Expressive writing therapy / journaling therapy | Macrobiotic diet | Shitake |
| Artichoke leaf | Eye Movement Desensitization and Reprocessing (EMDR) | Magnesium supplements | Shuanghuanglian (a Chinese herbal medicine) |
| Astragalus / Milkvetch (a Chinese herbal medicine) | Feldenkrais | Magnetic therapy / magnetic field therapy / biomagnetic therapy | Sidda medicine (a type of Indian (East Asian) traditional medicine) |
| Auricular acupuncture / ear acupuncture | Feng shui | Marijuana, marihuana / cannabis / cannabinoids / C. sativa / C. indica (exclude for purely psychoactive uses) | Soy / soybeans |
| Ayurveda / Ayurvedic medicine (a type of Indian (East Asian) traditional medicine) | Feverfew | Massage therapy | Speleotherapy |
| Bach flower remedies | Fish oil (omega-3 fatty acids) supplements | Meditation | Spinal manipulation |
| Baduanjin | Flor∙Essence formula / flower essences | Mediterranean diet | Spiritual healing |
| Balneotherapy | Folic acid / folate (Vitamin B9) supplements (many people would not include for prevention of neural tube defects) | Melatonin | St. John’s wort (Hypericum perforatum L.) |
| Bee stings / bee venom | Free and Easy Wanderer (a Chinese herbal medicine) | Mesotherapy | Structural integration |
| Beta-sitosterol (a component of saw palmetto) | Gamma-linolenic acid (GLA) (an omega-6 fatty acid) supplements | Milk thistle | Suxiao jiuxin wan (a Chinese herbal medicine) |
| Bibliotherapy | Garlic | Mindfulness | Tai chi / tai ji |
| Bioenergetics | Gerovital H3 (primary ingredient is procaine hydrochloride) | Moxibustion | Testosterone |
| Biofeedback | Gerson therapy | Music therapy / sound therapy | Therapeutic touch / healing touch |
| Biofunctional diagnostic testing | Gestalt therapy | Myofascial release | Thiamine (Vitamin B1) supplements |
| Biotin (Vitamin B7) supplements | Ginger | Naprapathy | Tianmadingxian capsule (a Chinese herbal medicine) |
| Botanical supplements | Ginkgo biloba | Nature therapy | Traditional African healing |
| Bovine cartilage | Ginseng | Naturopathy / naturopathic medicine | Traditional Arabic medicine |
| Breathing exercises in mind-body medicine (e.g., breathwork) (exclude for physical therapy, eg treatment of cystic fibrosis) | Glucosamine supplements | Neuromuscular therapy | Traditional Chinese medicine |
| Calcium supplements (many people would not include for prevention of osteoarthritis) | Glutamine supplements | Niacin / Nicotinamide/ Niacinamide (Vitamin B3) supplements | Traditional Indian medicine |
| Calendula | Gluten-free diet | Omega-3 fatty acids | Traditional Japanese medicine |
| Calorie restriction | Green tea / Camellia sinensus) | Osteopathic manipulation | Traditional Korean medicine |
| Carnitine supplements | Guiling pa’an wan (a Chinese herbal medicine) | Ozone therapy | Traditional Tibetan medicine |
| Cayenne | Hair analysis | Pantothenic acid (Vitamin B5) supplements | Transcranial magnetic stimulation (exclude for treatment of depression) |
| Chelation therapy (exclude for treatment of medically diagnosed heavy metal poisoning (eg, mercury or lead) and for medically diagnosed excess iron (eg, thalassemia)) | Helminth therapy / trichuris suis ova / Trichuris trichiura ova | Passiflora | Transcutaneous electrical stimulation |
| Chinese herbal medicine | Hemp oil | Peppermint | Trigger point myotherapy |
| Chiropractic manipulation | Herbal medicine / herbalism | Phytoestrogens | Tui na |
| Chitosan supplements | High-fiber diet | Phytomedicines / Phytotherapy | Ultrasound / ultrasonic therapy (exclude diagnostic ultrasound) |
| Chondroitin sulfate | Hippotherapy / equine-assisted therapy (exclude when physical therapy only) | Pilates | Unani medicine / Yunani medicine (a type of Arabic or Indian (East Asian) traditional medicine) |
| Clinical ecology | Holistic therapy / holistic medicine | Plant medicines | Valerian |
| Cold laser therapy | Homeopathy | Play therapy | Vega testing |
| Colon cleansing / colon irrigation | Homoharringtonine (HHT) (a plant alkaloid) | Prayer | Vegan diet |
| Color therapy / chromotherapy | Honey | Prebiotics | Vegetarian diet |
| Cranberry | Horse chestnut | Probiotics | Visualization techniques |
| Craniosacral massage / Craniosacral therapy | Huangqi (a Chinese herbal medicine) | Procaine (only when used for aging) | Vitamin A supplements |
| Dance therapy | Huperzine A (a Chinese herbal medicine) | Prolotherapy | Vitamin B complex supplements |
| Danshen (a Chinese herbal medicine) | Hydrazine sulfate | Propolis | Vitamin B12 supplements |
| Deep tissue bodywork | Hydrotherapy | Protein supplements | Vitamin C supplements |
| Dehydroepiandrosterone (DHEA) supplements | Hyperbaric oxygen therapy (exclude for treatment of burns, wounds or infections, radiation injury, embolism, decompression disorders or carbon monoxide poisoning) | Psychotherapies incorporating mindfulness | Vitamin D supplements |
| Dengzhanhua preparations (a Chinese herbal medicine) | Hypnosis / hypnotherapy | Psychotherapies incorporating spirituality | Vitamin E supplements |
| Devil’s claw | Imagery / guided imagery | Puerarin (a Chinese herbal medicine) | Vitamin K supplements |
| Devil’s nettle | Iridology | Pulsed electromagnetic field therapy (see Electromagnetic therapy) | Vojta method / Reflexlocomotion |
| Devil’s root / Siberian ginseng / acanthopanax senticosus / ci wu jia | Iron supplements | Pyridoxine / Pyridoxal / Pyridoxamine (Vitamin B6) supplements | White willow bark |
| Dianxianning pill (a Chinese herbal medicine) | Jin Li Da liquor (a Chinese herbal medicine) | Qi Gong / chi-kung | Xiaxingci granule (a Chinese herbal medicine) |
| Dietary supplements | Kampo (a type of traditional Japanese medicine) | Radiesthesia | Yarrow / Achillea millefolium extract |
| Dihomogammalinolenic acid (DGLA) (an omega-6 fatty acid) supplements | Kava | Reflexology | Yoga therapy |
| Dimethylaminoethanol / dimethylethanolamine / Deanol (DMAE) | Ketogenic diet | Reflexotherapy | Zero balancing |
| Docosahexaenoic acid (DHA) (an omega-3 fatty acid) supplements | Kinesiology / applied kinesiology | Reiki | Zhixian I pill (a Chinese herbal medicine) |
| Doman Delacato patterning therapy | Kneipp therapies | Relaxation techniques | Zinc supplements |
|  | Krestin / PSK / PSP (Coriolus Versicolor extracts) | Riboflavin (Vitamin B2) supplements | Zishen Tongli Jianonang (a Chinese herbal medicine) |
|  |  | Rolfing®Structural Integration | Zone therapy |
