# Supplementary material for: A scoping review of network meta-analyses assessing the efficacy and safety of complementary and alternative medicine interventions
Source: Syst Rev. 2020 Apr 30;9:97. doi: 10.1186/s13643-020-01328-3 (PMC7191816; doi:10.1186/s13643-020-01328-3)
Supplement: Supplementary file 4 — Additional file 4. The completed PRISMA-NMA Assessments for the included studies are provided [file 13643_2020_1328_MOESM4_ESM.docx]

**Additional File 4: PRISMA-NMA Assessments by Study**

| **First Author (Year)** | **PRISMA Items from Background and Methods: Adequately Reported?** | | | | | | | | | | | | | | | | | |
| --- | --- | --- | --- | --- | --- | --- | --- | --- | --- | --- | --- | --- | --- | --- | --- | --- | --- | --- |
|  | **1** | **2** | **3** | **4** | **5** | **6** | **7** | **8** | **9** | **10** | **11** | **S1** | **12** | **13** | **14** | **S2** | **15** | **16** |
| Imamura^27^ (2010) |  | Y | Y | Y |  | Y | Y | Y | Y | Y | Y | Y | Y | Y | Y |  |  | Y |
| Wandel^28^ (2010) |  | Y | Y | Y |  | Y | Y |  | Y | Y | Y | Y | Y | Y | Y | Y |  | Y |
| Anothaisintawee^29^ (2011) | Y | Y | Y | Y |  | Y | Y | Y | Y | Y |  |  | Y | Y |  |  | Y |  |
| Thakkinstian^30^ (2012) |  | Y | Y | Y |  | Y | Y | Y | Y | Y | Y |  |  | Y |  |  |  |  |
| Nüesch^31^ (2012) |  |  | Y | Y |  | Y | Y | Y | Y | Y | Y | Y | Y | Y | Y | Y |  | Y |
| Cawston^32^ (2013) |  | Y | Y | Y |  | Y | Y |  | Y | Y | Y | Y | Y | Y | Y |  |  | Y |
| Corbett^114^ (2013) |  | Y | Y | Y | Y | Y | Y | Y | Y | Y |  |  | Y | Y | Y | Y | Y | Y |
| Snedecor^33^ (2013) |  | Y | Y | Y |  | Y | Y | Y | Y | Y | Y |  | Y | Y | Y | Y |  | Y |
| Kriston^34^ (2014) |  | Y |  | Y | Y | Y | Y | Y | Y | Y | Y | Y | Y | Y | Y | Y | Y |  |
| Gerger^35^ (2014) |  | Y | Y |  |  | Y | Y | Y | Y | Y | Y | Y | Y | Y | Y | Y |  | Y |
| Griebeler^36^ (2014) | Y | Y | Y | Y | Y | Y | Y |  | Y | Y | Y |  | Y | Y | Y |  | Y | Y |
| Wang^37^ (2014) |  |  | Y | Y |  | Y | Y |  | Y | Y | Y |  | Y | Y |  | Y |  |  |
| Reinecke^110^ (2015) |  |  |  |  |  | Y | Y | Y | Y | Y | Y |  | Y | Y |  |  |  | Y |
| Lewis^38^ (2015) | Y | Y | Y | Y | Y | Y | Y | Y | Y | Y | Y | Y | Y |  | Y |  | Y | Y |
| Linde^39^ (2015) | Y | Y | Y | Y | Y |  | Y | Y | Y | Y | Y | Y | Y | Y |  | Y | Y | Y |
| Zeng^40^ (2015) | Y | Y | Y | Y |  | Y | Y | Y | Y |  | Y |  | Y | Y | Y | Y | Y | Y |
| Zhu^41^ (2015) | Y | Y | Y | Y |  | Y | Y |  | Y | Y | Y |  | Y | Y | Y | Y | Y |  |
| Dong^43^ (2015) | Y |  | Y |  |  | Y | Y | Y | Y | Y | Y |  | Y | Y |  | Y |  | Y |
| Devoe^44^ (2018) | Y | Y | Y |  | Y | Y | Y | Y | Y | Y | Y | Y | Y | Y | Y | Y | Y | Y |
| Slade^45^ (2018) |  | Y | Y | Y |  | Y | Y | Y | Y | Y | Y |  |  | Y | Y | Y |  |  |
| Zhu^42^ (2018) | Y | Y | Y |  |  | Y | Y |  | Y | Y | Y |  | Y | Y | Y | Y | Y | Y |
| Zhang^46^ (2018) |  |  |  |  |  | Y | Y |  | Y | Y |  |  | Y |  |  | Y | Y |  |
| van den Akker^47^ (2018) | Y | Y | Y |  | Y | Y | Y |  | Y | Y | Y | Y | Y | Y | Y | Y | Y |  |
| MacPherson^103^ (2017) |  | Y | Y | Y | Y | Y | Y | Y | Y | Y | Y |  | Y | Y | Y | Y | Y | Y |
| Howarth^104^ (2016) |  | Y | Y | Y | Y | Y | Y | Y | Y | Y | Y | Y | Y | Y | Y | Y |  |  |
| Grant^48^ (2015) |  | Y | Y | Y | Y | Y | Y | Y | Y | Y | Y |  | Y | Y | Y | Y | Y | Y |
| Wei^49^ (2018) | Y | Y | Y | Y |  | Y | Y | Y | Y | Y | Y | Y | Y | Y | Y | Y | Y | Y |
| Fu^50^ (2018) | Y | Y | Y | Y |  | Y | Y | Y | Y | Y | Y |  |  | Y | Y |  | Y |  |
| Freeman^51^ (2018) | Y | Y | Y | Y | Y |  | Y | Y | Y |  |  |  |  | Y | Y | Y |  | Y |
| Tsikopoulos^52^ (2018) | Y | Y | Y | Y | Y | Y | Y | Y | Y | Y | Y | Y | Y | Y | Y | Y | Y | Y |
| Lee^53^ (2018) | Y | Y | Y | Y |  | Y | Y |  | Y | Y | Y | Y |  | Y | Y | Y |  |  |
| Hilfiker^54^ (2018) | Y | Y | Y | Y | Y | Y | Y | Y | Y | Y |  |  | Y | Y | Y | Y | Y | Y |
| Xie^55^ (2018) | Y | Y | Y | Y | Y | Y | Y | Y | Y | Y | Y |  | Y | Y | Y | Y | Y |  |
| Cai^116^ (2017) | Y | Y | Y | Y | Y | Y | Y | Y | Y | Y |  |  | Y | Y | Y | Y | Y | Y |
| Pang^56^ (2018) | Y | Y | Y | Y | Y | Y | Y | Y | Y | Y |  |  | Y | Y | Y |  | Y | Y |
| Sarri^57^ (2017) | Y | Y | Y | Y | Y | Y | Y | Y | Y | Y | Y | Y | Y | Y | Y | Y |  | Y |
| Feng^58^ (2018) | Y | Y | Y | Y | Y | Y | Y | Y | Y | Y | Y |  | Y | Y | Y | Y | Y | Y |
| Yeh^59^ (2017) | Y | Y | Y | Y |  | Y | Y |  | Y | Y | Y |  | Y | Y | Y | Y | Y | Y |
| Gartlehner^60^ (2015) |  | Y | Y | Y | Y | Y | Y | Y | Y | Y | Y | Y | Y | Y | Y |  | Y | Y |
| Wen^61^ (2017) | Y | Y | Y | Y | Y | Y | Y | Y | Y | Y | Y |  | Y | Y | Y |  | Y | Y |
| Ma^113^ (2017) | Y | Y | Y | Y | Y | Y | Y | Y |  | Y | Y | Y | Y | Y | Y | Y | Y | Y |
| Khaing^62^ (2017) | Y | Y | Y | Y | Y | Y | Y | Y | Y | Y | Y |  | Y | Y | Y | Y | Y | Y |
| Zhang^107^ (2018) | Y | Y | Y | Y |  | Y | Y | Y | Y | Y | Y | Y | Y | Y | Y | Y | Y |  |
| Haggman-Henrikson^63^ (2017) | Y |  | Y | Y | Y | Y | Y | Y | Y | Y | Y |  | Y | Y | Y |  |  |  |
| Ho^64^ (2017) | Y |  | Y | Y |  | Y | Y | Y | Y | Y | Y |  | Y | Y | Y | Y |  |  |
| Muñoz^106^ (2017) | Y | Y | Y | Y |  | Y | Y | Y | Y | Y |  |  | Y | Y | Y | Y | Y | Y |
| Feng^65^ (2017) | Y | Y | Y | Y |  | Y | Y |  | Y | Y | Y |  | Y | Y | Y |  | Y | Y |
| Fu^66^ (2017) |  |  | Y | Y |  | Y |  |  | Y | Y | Y | Y | Y | Y | Y | Y | Y |  |
| Feng^67^ (2017) |  | Y | Y | Y |  | Y | Y | Y | Y | Y | Y | Y | Y | Y | Y |  |  |  |
| Wang^68^ (2017) | Y | Y | Y | Y |  | Y | Y |  | Y | Y | Y |  | Y | Y | Y | Y |  | Y |
| Yang^69^ (2017) |  |  | Y | Y |  | Y | Y |  |  | Y | Y |  | Y | Y | Y | Y | Y |  |
| Liang^70^ (2018) |  | Y | Y | Y |  | Y | Y |  | Y | Y | Y | Y | Y | Y | Y | Y | Y |  |
| Khan^71^ (2018) |  |  | Y | Y | Y | Y | Y |  |  | Y |  |  | Y | Y |  |  |  |  |
| Li^72^ (2017) |  | Y | Y | Y | Y | Y | Y | Y | Y | Y | Y | Y | Y | Y | Y | Y |  |  |
| Zhang^105^ (2017) |  | Y | Y | Y |  | Y | Y | Y | Y | Y | Y | Y | Y | Y | Y |  | Y | Y |
| Qin^73^ (2016) |  |  | Y | Y |  | Y | Y | Y | Y | Y | Y | Y | Y | Y | Y | Y |  | Y |
| Huang^74^ (2016) |  | Y | Y | Y |  | Y | Y |  |  | Y | Y | Y | Y | Y | Y | Y | Y |  |
| Skapinakis^75^ (2016) |  | Y | Y | Y | Y | Y | Y | Y | Y | Y | Y | Y | Y | Y | Y | Y |  | Y |
| Morrell^76^ (2016) |  | Y |  | Y | Y | Y | Y | Y | Y | Y | Y | Y | Y | Y | Y |  |  | Y |
| Kasatpibal^77^ (2017) | Y | Y | Y | Y |  | Y | Y |  |  | Y | Y |  | Y | Y | Y | Y | Y | Y |
| Woods^78^ (2017) |  | Y | Y | Y |  | Y | Y | Y |  |  |  |  | Y | Y | Y | Y |  | Y |
| Su^79^ (2017) | Y | Y | Y | Y | Y | Y | Y | Y | Y | Y |  |  | Y | Y | Y | Y |  | Y |
| Palmer^109^ (2016) |  | Y | Y | Y | Y | Y | Y |  | Y | Y |  |  | Y | Y | Y | Y |  | Y |
| Catala-Lopez^80^ (2017) | Y | Y | Y | Y | Y | Y | Y | Y | Y | Y | Y | Y | Y | Y | Y | Y | Y | Y |
| Westby^81^ (2017) |  | Y | Y | Y | Y | Y | Y | Y | Y | Y | Y | Y | Y | Y | Y | Y | Y | Y |
| Zhang^82^ (2017) |  | Y | Y | Y |  | Y | Y | Y | Y | Y | Y |  | Y | Y | Y | Y | Y | Y |
| Yu^83^ (2017) |  | Y | Y | Y |  | Y | Y |  |  | Y | Y |  | Y | Y |  |  | Y |  |
| van Nooten^84^ (2017) | Y | Y | Y | Y |  | Y | Y | Y |  | Y | Y |  | Y | Y | Y | Y | Y | Y |
| Wang^85^ (2017) |  | Y | Y | Y |  | Y | Y |  |  | Y | Y |  | Y | Y | Y |  | Y | Y |
| Sekercioglu^86^ (2017) | Y | Y | Y | Y | Y | Y | Y | Y | Y | Y | Y | Y | Y | Y | Y | Y | Y | Y |
| Amaral^87^ (2017) | Y |  | Y | Y | Y | Y | Y |  | Y |  | Y |  | Y | Y | Y | Y |  | Y |
| Zhang^88^ (2017) |  | Y | Y | Y |  | Y | Y |  | Y | Y | Y |  | Y | Y | Y | Y | Y |  |
| Dulai^89^ (2016) | Y | Y | Y | Y | Y | Y | Y | Y |  | Y | Y |  | Y | Y | Y | Y | Y | Y |
| Sawangjit^90^ (2016) | Y | Y | Y | Y | Y | Y | Y | Y |  | Y | Y |  | Y | Y | Y | Y | Y | Y |
| Wang^91^ (2016) | Y | Y | Y | Y | Y | Y | Y |  | Y | Y | Y |  | Y | Y | Y | Y | Y |  |
| Chung^92^ (2016) |  |  | Y | Y |  | Y | Y |  |  | Y |  |  | Y | Y | Y | Y | Y | Y |
| Pompoli^93^ (2016) |  | Y | Y | Y |  | Y | Y | Y | Y | Y | Y |  | Y | Y | Y | Y | Y | Y |
| Rochwerg^94^ (2016) |  | Y | Y | Y |  | Y | Y | Y | Y | Y |  |  | Y | Y | Y | Y | Y | Y |
| Wu^95^ (2016) | Y | Y | Y | Y |  | Y | Y |  | Y | Y | Y |  | Y | Y | Y |  | Y | Y |
| Linde^108^ (2016) |  | Y | Y | Y | Y | Y | Y |  | Y | Y | Y |  | Y | Y | Y |  |  | Y |
| Dong^96^ (2016) | Y | Y | Y | Y |  | Y | Y |  | Y | Y | Y |  | Y | Y | Y | Y |  | Y |
| Steenhuis^97^ (2015) |  | Y | Y | Y |  | Y | Y |  | Y |  | Y |  | Y | Y | Y | Y | Y | Y |
| Lehert^98^ (2015) |  |  | Y | Y |  | Y | Y | Y |  |  |  |  |  | Y | Y |  |  | Y |
| Dong^99^ (2015) |  | Y | Y | Y |  | Y | Y |  | Y | Y | Y | Y | Y | Y | Y | Y | Y | Y |
| Loveman^100^ (2015) |  | Y | Y | Y |  |  | Y | Y | Y | Y |  |  | Y | Y |  |  |  | Y |
| Kongtharvonskul^101^ (2015) | Y | Y | Y | Y |  | Y | Y | Y | Y | Y | Y |  | Y | Y | Y |  | Y |  |
| Di^111^ (2018) | Y | Y | Y | Y |  | Y | Y | Y | Y | Y | Y |  | Y | Y | Y | Y |  |  |
| Han^102^ (2017) |  | Y | Y | Y |  | Y | Y | Y | Y | Y | Y |  | Y | Y | Y | Y | Y | Y |
| Wei^112^ (2017) |  | Y | Y | Y |  | Y | Y |  |  | Y | Y |  |  | Y | Y | Y |  |  |

| **First Author (Year)** | **PRISMA Items from Results and Discussion: Adequately Reported?** | | | | | | | | | | | | | |
| --- | --- | --- | --- | --- | --- | --- | --- | --- | --- | --- | --- | --- | --- | --- |
|  | **17** | **S3** | **S4** | **18** | **19** | **20** | **21** | **S5** | **22** | **23** | **24** | **25** | **26** | **27** |
| Imamura^27^ (2010) | Y |  | Y | Y | Y | Y | Y |  |  | Y | Y | Y | Y | Y |
| Wandel^28^ (2010) |  | Y | Y | Y | Y | Y | Y | Y |  | Y | Y | Y | Y | Y |
| Anothaisintawee^29^ (2011) | Y | Y |  | Y | Y | Y | Y |  | Y | Y | Y | Y | Y | Y |
| Thakkinstian^30^ (2012) | Y | Y |  | Y |  | Y | Y |  |  |  | Y |  | Y |  |
| Nüesch^31^ (2012) | Y | Y | Y | Y |  |  | Y | Y |  | Y | Y | Y | Y | Y |
| Cawston^32^ (2013) | Y | Y |  | Y | Y | Y | Y |  |  | Y | Y | Y | Y |  |
| Corbett^114^ (2013) | Y | Y | Y | Y | Y | Y | Y | Y | Y | Y | Y | Y | Y | Y |
| Snedecor^33^ (2013) | Y | Y | Y | Y |  | Y | Y | Y |  | Y | Y | Y | Y |  |
| Kriston^34^ (2014) | Y | Y | Y | Y | Y | Y | Y | Y | Y | Y | Y | Y | Y | Y |
| Gerger^35^ (2014) | Y | Y | Y | Y | Y |  | Y | Y |  | Y | Y | Y | Y |  |
| Griebeler^36^ (2014) | Y | Y | Y | Y | Y |  | Y | Y |  | Y | Y |  | Y | Y |
| Wang^37^ (2014) | Y |  |  | Y |  |  | Y | Y |  |  | Y | Y | Y |  |
| Reinecke^110^ (2015) | Y |  | Y | Y | Y | Y | Y |  |  | Y | Y | Y | Y | Y |
| Lewis^38^ (2015) | Y | Y | Y | Y |  | Y | Y |  | Y | Y | Y | Y | Y | Y |
| Linde^39^ (2015) | Y | Y | Y | Y | Y |  | Y | Y | Y | Y | Y | Y | Y | Y |
| Zeng^40^ (2015) | Y | Y | Y | Y | Y |  | Y | Y | Y | Y | Y | Y | Y | Y |
| Zhu^41^ (2015) | Y | Y | Y | Y | Y |  | Y | Y | Y |  | Y | Y | Y |  |
| Dong^43^ (2015) | Y | Y |  | Y | Y |  | Y | Y |  | Y | Y | Y | Y | Y |
| Devoe^44^ (2018) | Y | Y | Y | Y | Y | Y | Y | Y | Y |  | Y | Y | Y | Y |
| Slade^45^ (2018) | Y | Y |  | Y | Y | Y | Y | Y | Y |  | Y | Y | Y | Y |
| Zhu^42^ (2018) | Y | Y | Y | Y | Y | Y | Y | Y | Y | Y | Y | Y | Y | Y |
| Zhang^46^ (2018) | Y | Y | Y | Y |  | Y | Y | Y |  |  | Y | Y | Y | Y |
| van den Akker^47^ (2018) | Y | Y | Y | Y | Y | Y | Y | Y | Y |  | Y | Y | Y | Y |
| MacPherson^103^ (2017) | Y | Y | Y | Y | Y | Y | Y | Y | Y | Y | Y | Y | Y | Y |
| Howarth^104^ (2016) | Y | Y | Y | Y | Y | Y | Y | Y |  |  | Y | Y | Y | Y |
| Grant^48^ (2015) | Y | Y | Y | Y | Y | Y | Y | Y | Y | Y | Y | Y | Y | Y |
| Wei^49^ (2018) | Y | Y | Y | Y | Y | Y | Y | Y | Y | Y | Y | Y | Y |  |
| Fu^50^ (2018) | Y | Y | Y | Y |  | Y | Y |  | Y |  | Y | Y | Y | Y |
| Freeman^51^ (2018) | Y | Y | Y |  |  | Y | Y |  |  | Y | Y | Y | Y | Y |
| Tsikopoulos^52^ (2018) | Y | Y |  | Y | Y | Y | Y |  |  | Y | Y | Y | Y |  |
| Lee^53^ (2018) | Y | Y | Y | Y |  | Y | Y |  |  | Y | Y | Y | Y | Y |
| Hilfiker^54^ (2018) | Y | Y | Y | Y | Y | Y | Y | Y |  | Y | Y | Y | Y |  |
| Xie^55^ (2018) | Y | Y |  | Y |  | Y | Y | Y | Y |  | Y | Y | Y | Y |
| Cai^116^ (2017) | Y | Y | Y | Y | Y | Y | Y | Y | Y | Y | Y | Y | Y | Y |
| Pang^56^ (2018) | Y | Y |  | Y | Y | Y | Y | Y | Y | Y | Y | Y | Y | Y |
| Sarri^57^ (2017) | Y | Y | Y | Y | Y | Y | Y | Y |  | Y | Y | Y | Y | Y |
| Feng^58^ (2018) | Y | Y | Y | Y | Y | Y | Y |  | Y |  | Y | Y | Y | Y |
| Yeh^59^ (2017) | Y | Y | Y | Y | Y | Y | Y | Y | Y | Y | Y | Y | Y |  |
| Gartlehner^60^ (2015) | Y | Y | Y | Y | Y | Y | Y |  |  | Y | Y | Y | Y | Y |
| Wen^61^ (2017) | Y | Y | Y | Y | Y | Y | Y |  | Y | Y | Y | Y | Y |  |
| Ma^113^ (2017) | Y | Y | Y | Y | Y | Y | Y |  | Y | Y | Y | Y | Y | Y |
| Khaing^62^ (2017) | Y | Y | Y | Y | Y | Y | Y | Y | Y | Y | Y | Y | Y | Y |
| Zhang^107^ (2018) | Y | Y |  | Y | Y | Y | Y |  | Y |  | Y |  | Y | Y |
| Haggman-Henrikson^63^ (2017) | Y | Y | Y | Y | Y | Y | Y |  |  |  | Y | Y | Y | Y |
| Ho^64^ (2017) | Y | Y | Y | Y | Y | Y | Y | Y | Y |  | Y | Y | Y |  |
| Muñoz^106^ (2017) | Y | Y | Y | Y | Y | Y | Y | Y | Y | Y | Y | Y | Y |  |
| Feng^65^ (2017) | Y | Y | Y | Y | Y | Y | Y |  | Y | Y | Y | Y | Y |  |
| Fu^66^ (2017) | Y | Y | Y | Y | Y | Y | Y | Y | Y |  | Y | Y | Y | Y |
| Feng^67^ (2017) | Y | Y | Y | Y | Y | Y | Y | Y |  |  | Y | Y | Y | Y |
| Wang^68^ (2017) | Y |  | Y | Y | Y | Y | Y | Y |  | Y | Y | Y | Y | Y |
| Yang^69^ (2017) | Y | Y |  | Y | Y | Y | Y |  | Y | Y | Y | Y | Y | Y |
| Liang^70^ (2018) | Y | Y | Y | Y | Y | Y | Y | Y | Y |  | Y | Y | Y |  |
| Khan^71^ (2018) | Y | Y | Y | Y | Y | Y | Y |  |  |  | Y | Y | Y | Y |
| Li^72^ (2017) | Y | Y |  | Y | Y | Y | Y | Y |  |  | Y | Y | Y | Y |
| Zhang^105^ (2017) | Y | Y |  | Y | Y | Y | Y |  | Y | Y | Y | Y | Y | Y |
| Qin^73^ (2016) | Y | Y |  | Y | Y | Y | Y | Y |  | Y | Y | Y | Y |  |
| Huang^74^ (2016) | Y | Y | Y | Y | Y | Y | Y | Y | Y |  | Y | Y | Y | Y |
| Skapinakis^75^ (2016) | Y | Y | Y | Y | Y | Y | Y | Y |  | Y | Y | Y | Y | Y |
| Morrell^76^ (2016) | Y | Y |  |  |  | Y | Y |  | Y | Y | Y | Y | Y | Y |
| Kasatpibal^77^ (2017) | Y | Y | Y | Y | Y | Y | Y | Y | Y | Y | Y | Y | Y | Y |
| Woods^78^ (2017) |  | Y |  | Y | Y | Y | Y |  |  | Y | Y | Y | Y | Y |
| Su^79^ (2017) | Y | Y |  | Y | Y | Y | Y | Y |  | Y | Y | Y | Y | Y |
| Palmer^109^ (2016) | Y | Y |  | Y | Y | Y | Y | Y | Y | Y | Y | Y | Y | Y |
| Catala-Lopez^80^ (2017) | Y | Y | Y | Y | Y | Y | Y | Y | Y | Y | Y | Y | Y | Y |
| Westby^81^ (2017) | Y | Y | Y | Y | Y | Y | Y | Y | Y | Y | Y | Y | Y | Y |
| Zhang^82^ (2017) | Y | Y |  | Y | Y | Y | Y | Y | Y |  | Y | Y | Y | Y |
| Yu^83^ (2017) | Y | Y |  | Y |  | Y | Y |  | Y |  | Y | Y | Y | Y |
| van Nooten^84^ (2017) | Y | Y |  | Y | Y | Y | Y | Y | Y |  | Y | Y | Y | Y |
| Wang^85^ (2017) | Y | Y |  | Y | Y | Y | Y |  | Y | Y | Y | Y | Y | Y |
| Sekercioglu^86^ (2017) | Y | Y | Y | Y | Y | Y | Y | Y | Y | Y | Y | Y | Y | Y |
| Amaral^87^ (2017) | Y | Y |  | Y | Y | Y | Y |  | Y |  | Y | Y | Y | Y |
| Zhang^88^ (2017) | Y | Y | Y | Y | Y | Y | Y | Y | Y |  | Y | Y | Y | Y |
| Dulai^89^ (2016) | Y | Y |  | Y | Y | Y | Y | Y | Y | Y | Y | Y | Y | Y |
| Sawangjit^90^ (2016) | Y | Y | Y | Y | Y | Y | Y | Y | Y | Y | Y | Y | Y |  |
| Wang^91^ (2016) | Y | Y |  | Y | Y | Y | Y | Y | Y |  | Y | Y | Y | Y |
| Chung^92^ (2016) | Y |  |  | Y | Y | Y | Y | Y | Y | Y | Y | Y | Y |  |
| Pompoli^93^ (2016) | Y | Y |  | Y | Y | Y | Y | Y | Y | Y | Y | Y | Y | Y |
| Rochwerg^94^ (2016) | Y | Y |  | Y | Y | Y | Y | Y | Y | Y | Y | Y | Y | Y |
| Wu^95^ (2016) | Y | Y |  | Y | Y |  | Y | Y | Y | Y | Y | Y | Y |  |
| Linde^108^ (2016) | Y | Y |  | Y | Y | Y | Y | Y |  |  | Y | Y | Y | Y |
| Dong^96^ (2016) | Y | Y |  | Y | Y | Y | Y | Y |  | Y | Y | Y | Y |  |
| Steenhuis^97^ (2015) | Y | Y |  | Y | Y |  | Y | Y | Y | Y | Y | Y | Y | Y |
| Lehert^98^ (2015) |  |  |  |  |  |  | Y | Y | Y |  | Y | Y | Y | Y |
| Dong^99^ (2015) | Y | Y |  | Y | Y | Y | Y | Y | Y | Y | Y | Y | Y | Y |
| Loveman^100^ (2015) | Y | Y |  | Y | Y | Y | Y |  |  | Y | Y | Y | Y | Y |
| Kongtharvonskul^101^ (2015) | Y | Y |  | Y | Y | Y | Y |  | Y |  | Y | Y | Y | Y |
| Di^111^ (2018) | Y | Y |  | Y | Y | Y | Y | Y |  |  | Y | Y | Y | Y |
| Han^102^ (2017) | Y | Y |  | Y | Y | Y | Y | Y | Y | Y | Y | Y | Y |  |
| Wei^112^ (2017) | Y | Y |  | Y | Y |  | Y |  |  |  | Y | Y | Y |  |
